# Study of the thermally-activated delayed fluorescence (TADF) mechanism of phenothiazine–dibenzothiophene-*S*,*S*-dioxide electron donor–acceptor dyads using steady-state and time-resolved optical and electron paramagnetic resonance spectroscopies

**DOI:** 10.1039/d5sc03644e

**Published:** 2025-09-24

**Authors:** Yuying Pei, Andrey A. Sukhanov, Xi Chen, Greta Sambucari, Laura Bussotti, Xin Liu, Jianzhang Zhao, Yanqin Li, Yanping Huo, Violeta K. Voronkova, Huimin Guo, Mariangela Di Donato

**Affiliations:** a State Key Laboratory of Fine Chemicals, Frontier Science Center for Smart Materials, School of Chemical Engineering, Dalian University of Technology Dalian 116024 P. R. China zhaojzh@dlut.edu.cn; b School of Chemistry, Dalian University of Technology Dalian 116024 P. R. China liyanqin@dlut.edu.cn; c Zavoisky Physical-Technical Institute, FRC Kazan Scientific Center of RAS Kazan 420029 Russia vor18@yandex.ru; d LENS (European Laboratory for Non-Linear Spectroscopy) Via N. Carrara 1 50019 Sesto Fiorentino (FI) Italy didonato@lens.unifi.it; e School of Chemistry, Dalian Key Laboratory of Intelligent Chemistry, Dalian University of Technology Dalian 116024 P. R. China guohm@dlut.edu.cn; f School of Chemical Engineering and Light Industry, Guangdong University of Technology Guangzhou 510006 P. R. China yphuo@gdut.edu.cn; g ICCOM-CNR Via Madonna del Piano 10-12 Sesto Fiorentino (FI) 50019 Italy

## Abstract

We studied a series of thermally activated delayed fluorescence (TADF) emitters based on phenothiazine–dibenzothiophene-*S*,*S*-dioxide (PTZ-DTO) electron donor–acceptor (D–A) dyads, using femtosecond/nanosecond transient absorption (fs/ns-TA) spectroscopy and pulsed laser-excited time-resolved electron paramagnetic resonance (TREPR) spectroscopy. In the analyzed compounds, a Se atom replaced the S atom in the phenothiazine (PTZ) unit to study the heavy atom effect on the reverse intersystem crossing (RISC). Moreover, oxidation of the PTZ to sulfoxide and sulfone was also used to tune the energy of the charge separated (CS) state, while keeping the other factors (^3^LE state energy, LE = locally excited, and electronic coupling between the donor and acceptor) intact to large extent. Fs-TA spectra show that charge separation occurs rapidly (*ca.* 7.6 ps) in non oxidized compunds, while for the compounds with the PTZ unit oxidized the CS is slightly slower (10.9 ps). Ns-TA spectroscopy demonstrated the coexistence of ^3^CS and ^3^LE states for the non oxidized dyads and the absence of heavy atom effect on RISC. The oxidation of the PTZ unit increased the CS state energy, so that only the ^3^LE state remains observed. All our results demonstrated that RISC rate constants are not enhaced in the presence of heavy atoms for the studied dyads. TREPR spectra show the presence of the ^3^LE state, and that the triplet state is formed most likely *via* spin–orbit charge transfer intersystem crossing (SOCT-ISC) because the electron spin polarization (ESP) phase pattern is (e, a, e, a, e, a). These studies are useful for an in-depth understanding of the photophysics of the electron D–A TADF emitters.

## Abstract 2

The photophysical mechanism of TADF emitters was studied using steady-state and time-resolved optical absorption/emission and electron paramagnetic resonance spectroscopy.

## Graphical abstract

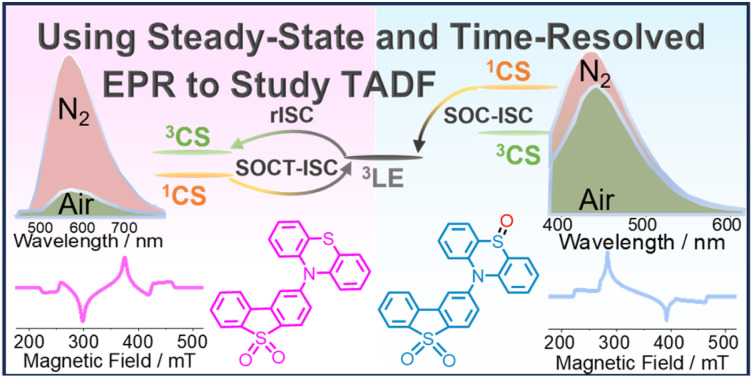

## Introduction

Electron donor–acceptor (D–A) dyads showing thermally-activated delayed fluorescence (TADF) properties have attracted notable attention because of their potential application in organic light emitting diodes (OLED).^1–16^ The most striking advantage of these compounds is that although mostly not containing heavy atoms (*e.g.*, Ir, Pt, Br, I, *etc.*), they are able to harvest triplet excitons and to populate the emissive singlet excited state *via* reverse intersystem crossing (RISC), thus emitting both prompt as well as the delayed fluorescence.^1,2^ The development of new TADF emitters and their application in OLED devices are very hot topics in current research, although a detailed understanding of the mechanisms of TADF, ISC, and RISC is missing in most cases.^4,5^

Reverse ISC and delayed fluorescence were initially explained in terms of a two-state model,^17–20^ according to which the triplet excited state (T_1_) and singlet excited state (S_1_) should have similar energy, such as to minimize the electron exchange energy (*J*) and the S_1_/T_1_ energy gap (Δ*E*_S_1_/T_1__), thus enhancing RISC, finally leading to TADF. A small Δ*E*_S_1_/T_1__ is beneficial for both ISC and RISC,^21^ but the electronic configuration of the involved S_1_ and T_1_ states are not unambiguously elucidated. In most cases the two states are assumed to be charge separated (CS) states and since the ^3^CS → ^1^CS transition is quantum mechanically forbidden,^22,23^ a simple two states model is often non-sufficient to rationalize the observation of delayed fluorescence. Recently, we have confirmed this postulation with several experiments.^24–27^ It was also proposed that the activation energy for the RISC is more important than the energy gap between the S_1_ and T_1_ states for observing TADF.^28^

In most cases, TADF emitters were only studied using steady state and time-resolved photoluminescence spectroscopies, but these techniques don't give direct experimental evidence for the existence of dark states such as ^3^CS and ^3^LE states (LE: locally excited).

Recently, it has been proposed that TADF should be rationalized in the frame of a three-state model,^23,29–37^ involving an emissive ^1^CS state, a dark ^3^CS state and a chromophore-localized ^3^LE dark state. The latter ^3^LE state is necessary to mediate the transition from the ^3^CS state to the emissive ^1^CS state (eqn (1)).^29,30^1
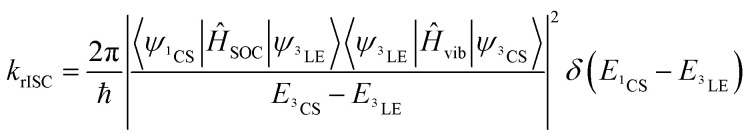


Theoretical studies have shown that for a well-known TADF emitter, PTZ-DBTO2, ISC between the ^1^CS and the ^3^CS state is driven by a hyperfine interaction of 0.2 cm^−1^, whereas the spin–orbit coupling between the singlet charge separated (^1^CT) state and the ^3^LE state is 2 cm^−1^, and the vibronic coupling between the ^3^CT and the ^3^LE is 65 cm^−1^.^29^ A theoretical study based on the spin-vibronic model predicts that the efficient RISC of PTZ-DBTO2 is due to the stronger S_1_/T_2_ coupling as compared to that between the S_1_/T_1_ states.^32^ However, experimental evidence confirming the co-existence of these three states is still scarce. Since the nature of the CS state of an electron D–A dyad depends on the electron dipolar interaction magnitude, the ion pairs can be considered either as free radical pairs (if the separation between the anion and the cation is large, *e.g.*, *r* > 30 Å) or as spin-correlated radical pairs (SCRP, where *r* ∼ 10–20 Å), or finally as ^3^CS state (with short separation between the anion and cation, for instance by a single bond, in this case the *J* value is large). All these species present similar bands in transient optical absorption spectra, which makes it difficult, if not impossible, to unambiguously discriminate between them only relying on optical spectroscopic methods.^25^

Recently femtosecond transient absorption (fs-TA) spectroscopy and nanosecond transient absorption (ns-TA) spectroscopy were used to characterize the photophysical processes of TADF emitters, evidencing the formation of ^1^LE or ^1^CS states as well as the ISC process.^25,38–42^ Experiments have shown that the rate of RISC can be significantly enhanced if the energy of ^3^LE state is similar to that of the ^3^CS state, thus contributing to efficient TADF.^24,43^ In contrast, the RISC process slows down significantly when ^3^LE and ^1^CS states with similar energies are missing, making TADF less efficient.^26,44,45^ Both fs-TA and ns-TA spectroscopies have provided direct experimental evidence for the spin-vibronic coupling mechanism,^29,30^ being able to detect dark states such as ^3^CS and ^3^LE states.

A discrimination between ^1^CS and ^3^CS states can be made based on their lifetime and emissive properties. In previous studies, we have confirmed that if the energy gap between the ^3^CS and ^3^LE states is large, the ^3^CS → ^1^CS transition is too slow to induce any TADF.^24,26,27^ Another useful technique, able to selectively detect the transient paramagnetic species involved in the TADF processes, such as the ^3^LE and ^3^CS states, as well as their interconversion and the ISC mechanism, is the pulsed laser excited time-resolved electron paramagnetic resonance (TREPR) spectroscopy.^46,47^ For instance, using TREPR spectroscopy it was proposed that for the carbazole-derived TADF compounds (4Cz-IPN), ISC occurs through a radical pair ISC (RP-ISC) mechanism, based on the electron spin polarization (ESP) of the ^3^LE state.^48^ Another report, analyzing the 4-(3,6-di-*tert*-butyl-9*H*-carbazol-9-yl)diphenyl sulfone (DTCz-DPS) TADF emitter through TREPR spectroscopy, showed that vibrations play a critical role for ISC.^49^ Evans *et al.* also found that a prominant ^3^CS state is present in case of good TADF emitters. Generally, the ^3^CS state has a significantly smaller zero field splitting (ZFS) *D* parameter than the ^3^LE state, because the average distance between the electrons is usually larger (point-dipole approximation).^43,48^ We used TREPR spectroscopy to study the photophysical mechanism of the TADF process, observing for instance both a ^3^LE state and a ^3^CS state in a naphthalimide–phenothiazine (NI-PTZ) dyad, which could be distinguished on the basis of their electron spin polarization pattern.^43^ We observed that the electron spin dipolar interaction is much stronger for ^3^CS states compared to SCRP states.^43,50,51^ This arises from the short distance between the electron donor and acceptor in NI-PTZ dyads,^49,52^ allowing us to observe the interconversion between the ^3^CS and ^3^LE states, which is a strong direct experimental evidence for the spin-vibronic coupling model of TADF.

**Scheme 1:**
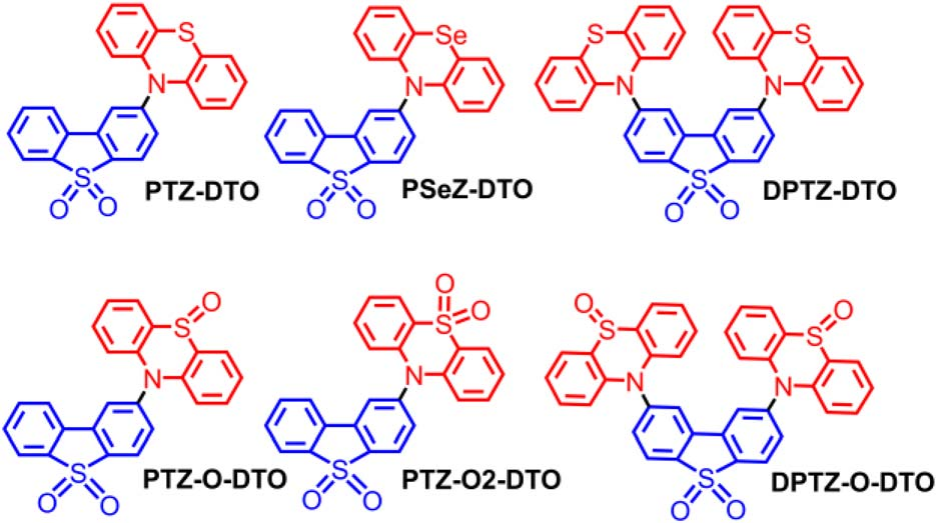
Molecular structures of the TADF emitters and reference compounds used in the study. The red units are the electron donors, and the blue units are the electron acceptors.

Although some evidence is present in the literatures, more experimental studies are needed to verify the above observations. Herein we selected a series representative TADF emitters based on the electron D–A couple phenothiazine–dibenzothiophene-*S*,*S*-dioxide (PTZ-DTO, Scheme 1).^53,54^ The photophysical properties of the compounds were studied by using steady state UV/vis absorption and fluorescence spectroscopy, and their excited state dynamics was analysed with fs-TA and ns-TA spectroscopy. Furthermore, TREPR spectroscopy was used for characterizing the transient paramagnetic species and their electron spin dynamics.

## Results and discussion

### Molecular structure designing rationales

We selected the D–A TADF emitters PTZ-DTO and DPTZ-DTO, based on phenothiazine (PTZ) as the electron donor, because of its strong electron-donating ability (*E*_OX_ = 0.18 V, *vs.* Fc/Fc^+^),^55^ and dibenzothiophene-*S*,*S*-dioxide (DTO) as the electron acceptor. The TADF properties of these two compounds have been previously reported and their application in polymer light emitting diodes OLED devices has been studied.^53,56^ However, details on the ISC and RISC mechanisms were not reported.

We then tuned the redox potential of the compounds by oxidizing the sulfur atom into sulfoxide and sulfone (PTZ-O-DTO, PTZ-O2-DTO, DPTZ-O-DTO), changing the electron-donating ability of PTZ, thus altering the energy of the CS state and consequently the TADF properties.^57^ We also substituted the sulfur (S) atom in the PTZ unit with selenium (Se), to change the photophysical properties of the dyads due to the introduction of a heavy atom (PSeZ-DTO).^58^ Previous studies have shown that the introduction of Se atoms in TADF emitters enhance the RISC, which is beneficial for OLED devices.^58–61^ The molecular structure of the compounds has been confirmed by ^1^H NMR, ^13^C NMR and HRMS characterizations.

### UV/vis absorption and fluorescence spectra

**Fig. 1:**
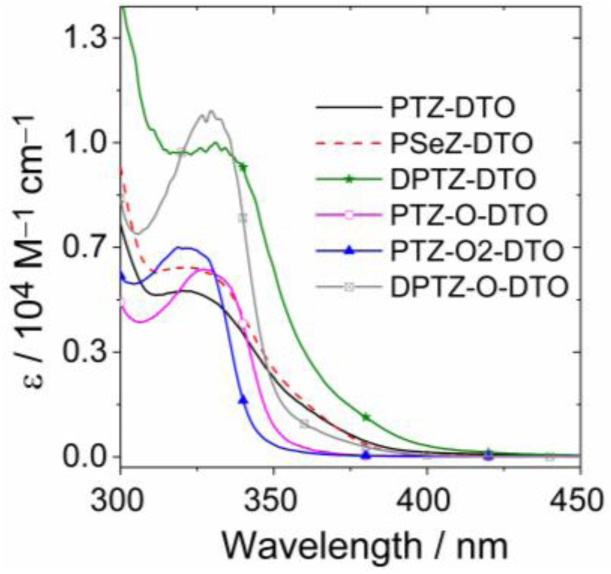
UV/vis absorption spectra of the compounds in dichloromethane (DCM). *c* = 3.0 × 10^−4^ M, 25 °C.

The UV/vis absorption spectra of the compounds in DCM are presented in Fig. 1. All compounds absorb in the 300–420 nm spectral range. The absorption spectra of PTZ and DTO have been previously reported, and their absorption bands are centered at *ca.* 320 nm.^54^ Since the HOMO and LUMO molecular orbitals have poor spatial overlap (SI), the absorption bands of PTZ-DTO and DPTZ-DTO result from the superposition the PTZ and DTO absorption bands, indicating negligible interaction between the electron donor and acceptor at the ground state. The absorption spectrum of PSeZ-DTO is similar to that of PTZ-DTO, although the absorption band centered at 320 nm is slightly stronger. For PTZ-DTO, PSeZ-DTO and DPTZ-DTO, a weak CT shoulder absorption band was observed. For PTZ-O-DTO, PTZ-O2-DTO and DPTZ-O-DTO, the absorption peaks slightly shift but remain in the 300–375 nm spectral range. For PTZ-O-DTO and PTZ-O2-DTO the CT absorption is hardly observed, even at high concentrations. It has previously been reported that due to the weak yet finite coupling between the DTO and PTZ moieties, there is an absorption band centered at 450 nm, assigned to the S_0_ → ^1^CT transition.^53,62,63^ For DPTZ-O-DTO, the CT absorption band is blue shifted and becomes weaker, indicating that the coupling between the electron donor and acceptor is weaker after oxidation of the PTZ unit. We confirmed that these compounds are photostable with continuous photo-irradiation experiments.^64^

**Fig. 2:**
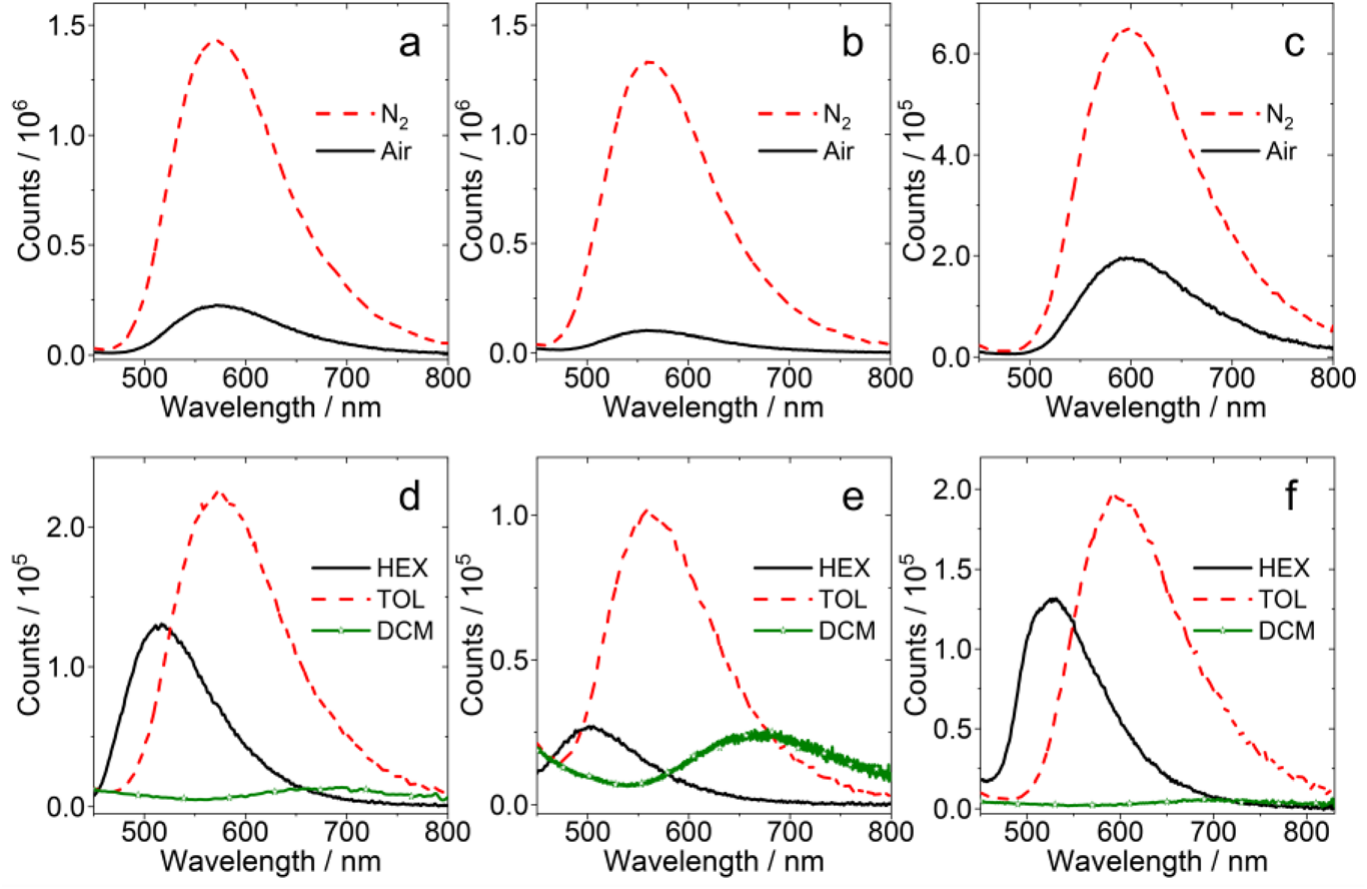
Fluorescence emission spectra of the compounds under N_2_ and air atmosphere of (a) PTZ-DTO, (b) PSeZ-DTO and (c) DPTZ-DTO in toluene (TOL). Fluorescence emission spectra of the compounds in different solvents of (d) PTZ-DTO, (e) PSeZ-DTO and (f) DPTZ-DTO in aerated solution. Optically-matched solutions were used, *A* = 0.1, *λ*_ex_ = 310 nm, 25 °C.

The fluorescence spectra of the compounds were studied (Fig. 2). The spectra of PTZ-DTO and DPTZ-DTO are similar to those reported previously.^53,62,63^ For all the three compounds PTZ-DTO, PSeZ-DTO and DPTZ-DTO, a broad and structureless emission band in the range of 500–800 nm is observed, attributed to the ^1^CT → S_0_ emission. However, the fluorescence emission wavelength and intensity of the three compounds are different. For PTZ-DTO, the fluorescence band is centered at 568 nm (Fig. 2a), for PSeZ-DTO it is centered at 559 nm (Fig. 2b), while it is centered at 595 nm for DPTZ-DTO (Fig. 2c).

We then measured the fluorescence of the compounds under different atmospheres, finding that it is significantly enhanced under N_2_ atmosphere and quenched by O_2_ under air atmosphere, indicating the involvement of triplet states in the emission process.^65,66^ Greatly enhanced fluorescence under N_2_ is typical for compounds showing TADF.^1^ The luminescence of PTZ-O-DTO, PTZ-O2-DTO and DPTZ-O-DTO, in which the PTZ unit is oxidized, were not significantly quenched under air atmosphere (Fig. S8), suggesting that the TADF properties of the oxidized compounds is not significant.^26,67^

We also measured the fluorescence spectra of the compounds in different solvents (Fig. 2d–f). For PTZ-DTO, PSeZ-DTO and DPTZ-DTO, the emission bands are red-shifted with increased solvent polarity. The emission intensity in TOL is stronger than that in hexane (HEX), which is often observed in case of CS state emission.^65,66,68,69^ The fluorescence bands of PTZ-O-DTO, PTZ-O2-DTO and DPTZ-O-DTO in different solvents are also significantly red-shifted, but the fluorescence intensity is not significantly quenched, indicating that the emissive state might not be a conventional intramolecular charge transfer state (Fig. S8).

### Fluorescence lifetime studies

**Fig. 3:**
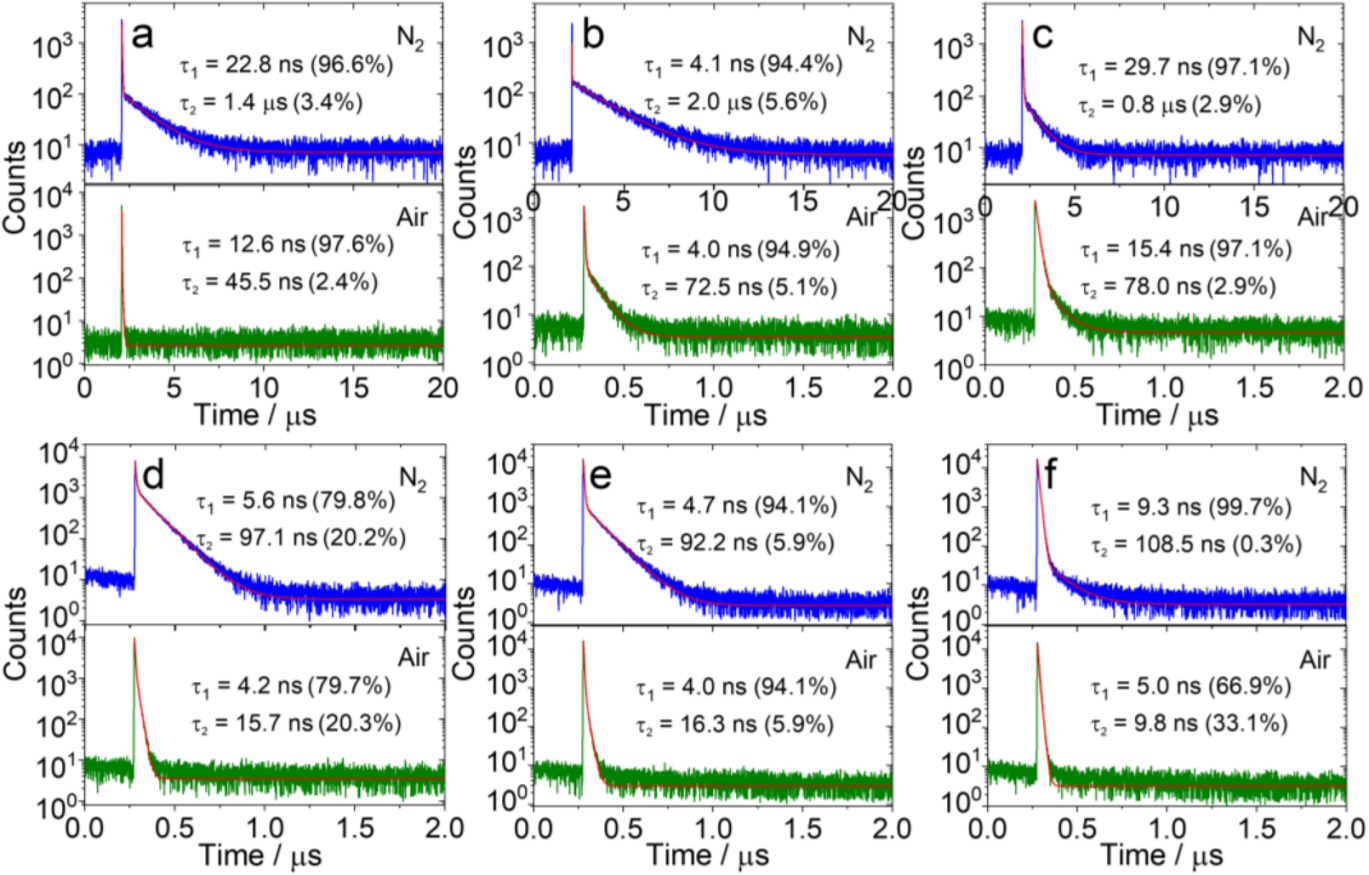
Fluorescence decay traces of (a) PTZ-DTO at 560 nm, (b) PSeZ-DTO at 550 nm, (c) DPTZ-DTO at 580 nm, (d) PTZ-O-DTO at 400 nm, (e) PTZ-O2-DTO at 410 nm and (f) DPTZ-O-DTO at 470 nm under different atmospheres (N_2_ and air). *λ*_ex_ = 340 nm, *c* = 1.0 × 10^−5^ M in TOL, 25 °C.

The luminescence lifetime of the TADF emitters in TOL under air and N_2_ atmospheres are compared in Fig. 3. The lifetime of the CS emission was monitored at 560 nm (Fig. 3a) for PTZ-DTO under N_2_ atmosphere, and it displayed a biexponential decay, with two lifetimes of 22.8 ns (96.6%) and 1.4 μs (3.4%), respectively. The short-lived component is assigned as prompt fluorescence, while the long-lived component as delayed fluorescence.^1^ In aerated solution, the lifetime of the delayed fluorescence is significantly shortened (45.5 ns), indicating that the triplet state is involved in the luminescence.^2,70–72^ The fluorescence decay of PTZ-DTO in the solid matrix Zeonex® COP 480R has been previously reported, also showing a double exponential decay.^53^

Fig. 3b presents the fluorescence lifetime of PSeZ-DTO monitored at 550 nm. Similar to PTZ-DTO, the emission of PSeZ-DTO presents a biexponential decay in N_2_ atmosphere: the lifetime of prompt fluorescence is 4.1 ns (94.4%) and that of delayed fluorescence is 2.0 μs (5.6%). Contrarily to what observed previously,^58,61,73^ in our case the presence of a heavy atom does not shorten the lifetime of the delayed fluorescence, suggesting that the heavy atom effects don't enhance significantly the rate of RISC in this compound. For PSeZ-DTO, the delayed fluorescence lifetime is shortened to 72.5 ns in aerated solution. We attribute the lack of heavy atom effect on the TADF property to the varied CS state energy upon the one-atom alternation of the molecular structure (see the Electrochemistry section).

The fluorescence decay traces of DPTZ-DTO is presented in Fig. 3c. Compared with PTZ-DTO, we observed a decreased lifetime for the slow decaying phase, which results 0.8 μs in the deaerated solution, while the lifetime of the fast decay component is 29.7 ns, longer than that of the PTZ-DTO. In this case the long lifetime is shortened under air atmosphere, indicating that the compound has TADF properties. The fluorescence of this compound behaves similarly to that of DPTZ-DTO (*τ*_PF_ = 33.0 ns and *τ*_DF_ = 1.1 μs).^74^

We also studied the luminescence lifetimes of PTZ-O-DTO and PTZ-O2-DTO (Fig. 3d and e), finding also in this case a biexponential decay. Under N_2_ atmosphere, the delayed fluorescence lifetime was determined 97.1 ns for PTZ-O-DTO and 92.2 ns for PTZ-O2-DTO, and both compounds displayed a high singlet oxygen quantum yield (*Φ*_Δ_. Tables 1 and 2). For DPTZ-O-DTO (Fig. 3f), the delayed fluorescence lifetime component accounts for very little and is almost negligible.

**Table 1:** Photophysical data of the compounds

Compounds	*λ*_abs_/nm^a^	*ε* b	*λ*_em_/nm^c^	*τ*_F_/ns^d^	*Φ*_F_ e (%)	*Φ*_Δ_ f (%)
PTZ-DTO	319	0.5	568	22.8 (96.6%)	6.1	0.2
				1400 (3.4%)		
PSeZ-DTO	322	0.6	559	4.1 (94.4%)	3.2	12.1
				2000 (5.6%)		
DPTZ-DTO	324	1.0	595	29.7 (97.1%)	6.5	10.0
				800 (2.9%)		
PTZ-O-DTO	325	0.6	439	5.6 (79.8%)	4.5	59.0
				97.1 (20.2%)		
PTZ-O2-DTO	321	0.7	405	4.7 (94.1%)	5.7	69.2
				92.2 (5.9%)		
DPTZ-O-DTO	330	1.1	470	9.3 (99.7%)	5.6	64.6
				108.5 (0.3%)		

a Maximal UV/vis absorption wavelength, *c* = 3.0 × 10^−4^ M, 25 °C.

b Molar absorption coefficient at absorption maxima, *ε*: 10^4^ M^−1^ cm^−1^, in DCM.

c Emission wavelength.

d Fluorescence lifetime, *λ*_ex_ = 340 nm.

e Fluorescence quantum yields, *λ*_ex_ = 310 nm.

f Singlet oxygen quantum yields, Ru(bpy)_3_[PF_6_]_2_ was used as standard compound (*Φ*_Δ_ = 57% in DCM), *λ*_ex_ = 343 nm.

**Table 2:** Singlet oxygen quantum yields (*Φ*_Δ_ in %) of the compounds in different solvents^a^

Compounds	HEX	TOL	DCM	ACN	MeOH
PTZ-DTO	11.7	0.2	—b	—b	—b
PSeZ-DTO	9.6	12.1	—b	—b	—b
DPTZ-DTO	0.3	10	—b	—b	—b
PTZ-O-DTO	28.7	59.0	86.5	70.8	11.7
PTZ-O2-DTO	4.8	69.2	99.2	99.5	41.7
DPTZ-O-DTO	—b	64.6	35.2	53.8	0.6

a The *E*_T_ (30) values of the solvents are 31.0 kcal mol^−1^ (HEX), 33.9 kcal mol^−1^ (TOL), 40.7 kcal mol^−1^ (DCM), 45.6 kcal mol^−1^ (ACN) and 55.4 kcal mol^−1^ (methanol (MeOH)), respectively. *Φ*_Δ_ with Ru(bpy)_3_[PF_6_]_2_ as standard (*Φ*_Δ_ = 0.57 in DCM) in different solvents, *λ*_ex_ = 330 nm.

b Not observed.

The photophysical data of all compounds are summarized in Table 1. The fluorescence quantum yields (*Φ*_F_) of the compounds are low, generally between 3 and 6%, indicating that non-radiative relaxation channels are quite efficient for all of them in fluid solution. We also measured the *Φ*_Δ_ of the compounds in different solvents to characterize the ISC efficiency. For PTZ-DTO, PSeZ-DTO and DPTZ-DTO we observed production of ^1^O_2_ only in low polar solvents, possibly because in polar media the CS state energy becomes lower than that of the ^3^LE state, thus preventing ^1^O_2_ production. Upon oxidation of the PTZ unit, the *Φ*_Δ_ of all compounds increases, especially in highly polar solvents. In this case the lowest energy triplet state is ^3^LE, according to the results obtained with ns-TA spectroscopy (see later section).

### Electrochemical properties

**Fig. 4:**
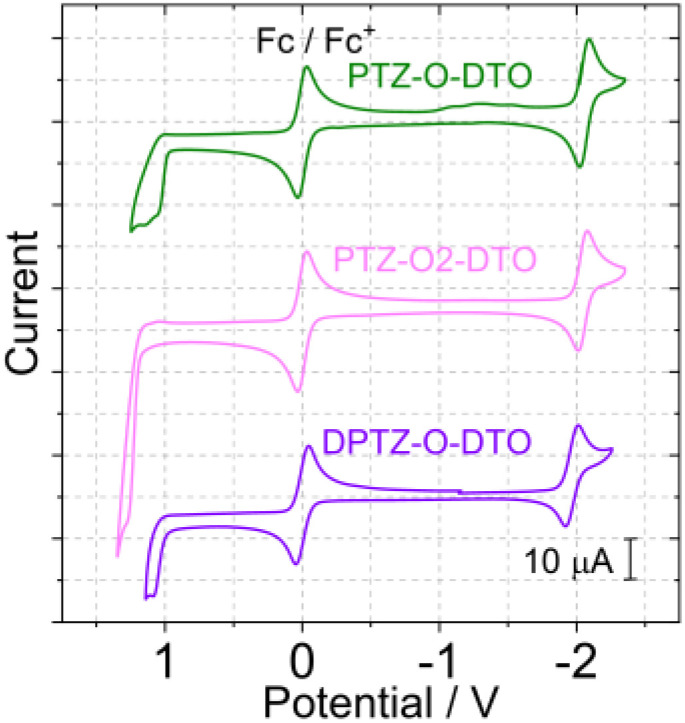
Cyclic voltammograms of the compounds PTZ-O-DTO, PTZ-O2-DTO recorded in deaerated acetonitrile (ACN), DPTZ-O-DTO was studied in deaerated DCM. Ferrocene (Fc) was used as internal reference (set as 0 V in the cyclic voltammograms), 0.10 M Bu_4_NPF_6_ as supporting electrolyte. Scan rates: 50 mV s^−1^, *c* = 1.0 × 10^−4^ M, 25 °C.

The redox potentials of the compounds were measured with cyclic voltammetry (Fig. 4) and the data are compiled in Table 3. For the compounds PTZ-O-DTO, PTZ-O2-DTO and DPTZ-O-DTO the oxidation waves are irreversible and significantly stronger if compared with the unoxidized compounds, because of the weakened electron donating ability of the PTZ moiety after oxidation.^55,57,75^ The free energy changes for the intramolecular electron transfer process can be calculated using the Rehm–Weller equation. The energies of the CS states (*E*_CS_) can be calculated with the eqn (2).^76–78^

**Table 3:** Redox potentials of the compounds in different solvents^a^

Compounds	*E*_OX_ (V)	*E*_RED_ (V)
PTZ-DTO^b^	+0.37	−2.12
PSeZ-DTO^b^	+0.45	−2.17
DPTZ-DTO^c^	+0.41	−2.15
PTZ-O-DTO^b^	+1.14	−2.05
PTZ-O2-DTO^b^	+1.28	−2.04
DPTZ-O-DTO^c^	+1.09	−1.96

a Cyclic voltammetry in N_2_-saturated solvents containing 0.10 M Bu_4_NPF_6_, glassy carbon electrode as the working electrode, Pt electrode as the counter electrode, Ag/AgNO_3_ couple as the reference electrode and ferrocene (Fc/Fc^+^) as internal reference (set as 0 V in the cyclic voltammograms).

b Measured in ACN.

c Measured in DCM.

A negative Gibbs free energy change (Δ*G*_CS_) of the compounds in polar solvents indicates thermodynamically allowed charge separation.^66,76,79–81^ In the non-polar solvent *n*-hexane CS is thermodynamically inhibited. As the polarity of the solvent increases, the CS state energy decreases.^82^ As noticed from Table 4, the energy of the CS state increases after the oxidation of PTZ but still decreases with the increasing solvent polarity. Based on the electrochemical and fluorescence emission data, we found that the energy of the CS state changed after the introduction of Se atom, resulting in changes in the S_1_ and T_1_ energy gaps. The change in the energy level of the CS state reasonably explains why Se substitution failed to significantly improve RISC. The energy of the ^3^LE state of the compounds can be obtained from the 77 K phosphorescence spectrum. The ^3^LE state energy of PTZ-DTO and PSeZ-DTO is approximated as 2.71 eV. It was found the ^3^CS and ^3^LE states have similar energy in toluene (^3^LE state of DPTZ-DTO is 2.67 eV), thus TADF is expected, and indeed it was observed. In polar solvents, the ^3^CS/^3^LE states energy gap becomes larger, and no TADF was observed. The three compounds in which the PTZ unit is oxidized have a large energy gap between the ^3^LE state and the CS state, therefore it is unlikely to observe significant TADF. Interestingly, the CS state energy varied noticeably with one-atom alternation (Se *vs.* S), which may impose substantial effects on the TADF property. Therefore, one should be careful in predicting heavy atom effects in TADF emitters.^80^

2 *E*_CS_ = *e*[*E*_OX_ − *E*_RED_] + Δ*G*_S_

3 Δ*G*^0^_CS_ = *e*[*E*_OX_ − *E*_RED_] − *E*_00_ + Δ*G*_S_

4
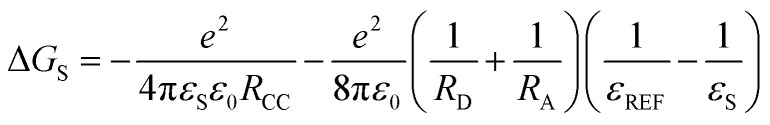


**Table 4:** Gibbs free energy changes for charge separation (Δ*G*_CS_) and charge separated states energy (*E*_CS_) of the compounds in different solvents^a^

	Δ*G*_CS_ (eV)					*E*_CS_ (eV)				
	HEX	TOL	DCM	MeOH	ACN	HEX	TOL	DCM	MeOH	ACN
PTZ-DTO^b^	−0.77	−0.94	−1.44	−1.57	−1.58	+2.90	+2.73	+2.23	+2.10	+2.09
PSeZ-DTO^c^	−0.70	−0.86	−1.31	−1.42	−1.43	+2.95	+2.79	+2.34	+2.23	+2.22
DPTZ-DTO^d^	−0.60	−0.79	−1.32	−1.46	−1.47	+3.03	+2.84	+2.31	+2.17	+2.16
PTZ-O-DTO^e^	+0.01	−0.18	−0.70	−0.84	−0.85	+3.64	+3.46	+2.94	+2.80	+2.79
PTZ-O2-DTO^f^	+0.03	−0.14	−0.64	−0.76	−0.77	+3.73	+3.56	+3.06	+2.93	+2.93
DPTZ-O-DTO^g^	−0.18	−0.35	−0.84	−0.97	−0.98	+3.46	+3.29	+3.80	+2.67	+2.66

a Cyclic voltammetry was recorded in deaerated solutions containing 0.10 M Bu_4_NPF_6_. Pt electrode is the counter electrode, glassy carbon electrode is the working electrode, and Ag/AgNO_3_ couple is the reference electrode.

b *E*_00_ = 3.67 eV.

c *E*_00_ = 3.65 eV.

d *E*_00_ = 3.63 eV.

e *E*_00_ = 3.64 eV.

f *E*_00_ = 3.70 eV.

g *E*_00_ = 3.64 eV. *E*_00_ (*E*_00_ = 1240/*λ*) is the singlet state energy of the compounds, *λ* is the wavelength of the crossing point between the normalized UV/vis absorption spectrum and the fluorescence spectrum.

### Femtosecond transient absorption spectra

**Fig. 5:**
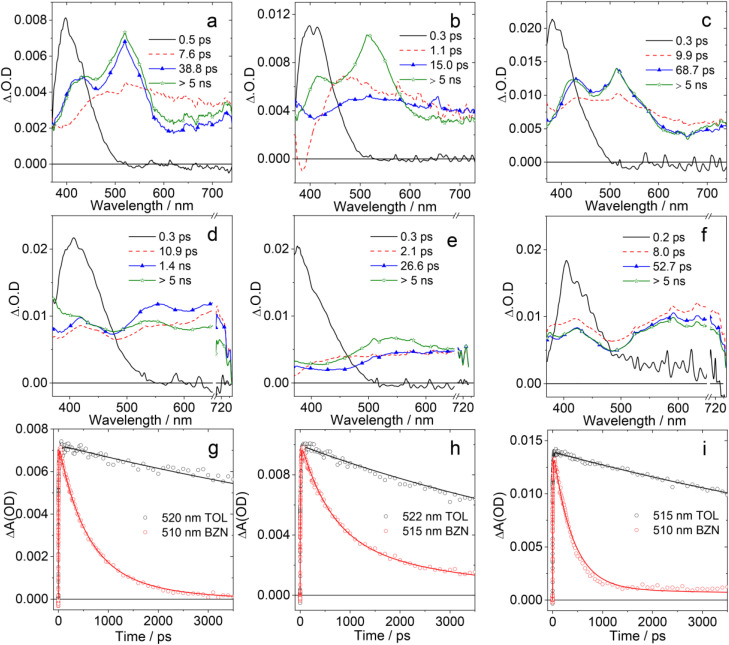
EADS obtained from global analysis of the transient absorption data recorded for (a) PTZ-DTO, (b) PSeZ-DTO, (c) DPTZ-DTO, (d) PTZ-O-DTO, (e) PTZ-O2-DTO and (f) DPTZ-O-DTO in TOL upon excitation at 350 nm. Panels (g)–(i) report a comparison of the kinetic traces recorded at the maximum of the PTZ^+^˙ signal for the three compounds presented in (a)–(c) in TOL and benzonitrile (BZN). The compounds were excited at 350 nm.

We analyzed the photoinduced behavior of the compounds by measuring their transient absorption spectra with 150 fs time resolution (Fig. 5). All compounds were excited at 350 nm, and transient data were acquired in a time interval spanning up to 3.6 ns. The kinetic traces were then fitted using a global analysis procedure and applying a linear unidirectional decay scheme. The fitting procedure allowed us to extract the time constants describing the excited state evolution and the associated spectral components, termed Evolution Associated Difference Spectra (EADS).^83,84^ The EADS obtained by analyzing the data recorded for the compounds PTZ-DTO, PSeZ-DTO and DPTZ-DTO in TOL solution are presented in Fig. 5.

The spectra of all compounds are qualitatively similar, although some difference can be noticed in the kinetic constants extracted from the fitting. In all cases the initial EADS, living less than 0.5 ps, shows an intense peak centered at about 400 nm. According to previous literature,^85^ this component is attributed to the response of the solvent, specifically to its two-photon absorption, which is promoted upon excitation with UV light. The following spectral component (red EADS in Fig. 5) appears as a very broad positive band, extending within all the probed spectral interval, for all samples.^86,87^

Based on the transient spectrum of the reference compound Br-DTO (Fig. S13), we attribute this component to the S_1_ singlet excited state of the compounds. For PTZ-DTO this state evolves in about 7.6 ps. The following EADS present two peaks, one centered at about 420 nm and the other at about 520 nm. Based on the results obtained from spectroelectrochemistry, these bands can be respectively assigned to the DTO^−^˙ anion and the PTZ^+^˙ cation, thus indicating the occurrence of photoinduced CS (Fig. S16).

The intensity of the signal slightly decreases on the following 38.8 ps timescale, and on this same timescale the band at 420 nm experiences a small blue shift. We attribute this evolution to a stabilization of the CS state induced by solvent rearrangement and vibrational cooling. The lifetime of the CS state is longer than the timescale accessed with our setup, indicating that charge recombination (CR) is expected to occur on a timescale longer than 5 ns.

Photoinduced CS was also observed for both PSeZ-DTO (Fig. 5b) and DPTZ-DTO (Fig. 5c). For the compound containing Se, the dynamics of CS appears a bit slower, in agreement with the modified oxidation potential of the electron donor occurring upon substituting S with Se. For this sample we observe a fast 1 ps component, attributed to a solvent induced relaxation of the S_1_ state, followed by CS, which occurs within 15 ps. CR is not observed within the investigated timescale also in case of PSeZ-DTO. The dynamics of DPTZ-DTO (Fig. 5c) is very similar to that of PTZ-DTO. For this sample, CS occurs in 9.9 ps and CR is expected to occur in >5 ns.

Fs-TA measurements were then repeated in a more polar solvent, benzonitrile (BZN) (Fig. S14), where the samples are quite soluble. In all cases we observed the occurrence of photoinduced CS. In the polar medium the lifetime of the CS state is shortened, as expected because of its stabilization in a more polar environment. Although a complete CR is still not observed, the cation and anion bands significantly decrease in intensity within the investigated time interval, suggesting an increased CR rate, as shown from the comparison of kinetic traces reported in Fig. 5.

Finally, we measured the transient spectra of the oxidized compounds PTZ-O-DTO, PTZ-O2-DTO and DPTZ-O-DTO in TOL, whose EADS are also reported in Fig. 5. The experimental results showed that narrower peaks were observed at 420 nm for these three compounds, while broader absorption bands were observed at 550 nm, and the spectral shapes were slightly different from those of the unoxidized compounds. For both PTZ-O-DTO and PTZ-O2-DTO, the final EADS presents an increased intensity in the short wavelength region (<400 nm), indicating the transition towards the localized triplet state, which absorbs at 350 nm, as evidenced by measurements performed on a longer timescale (see next paragraph). Measurements were repeated in BZN also for these samples. The transient absorption spectral characteristics of the oxidized compounds are similar to those observed in TOL, but there are some differences in the time constants of the kinetic processes (Fig. S15).

### Nanosecond transient absorption spectra

**Fig. 6:**
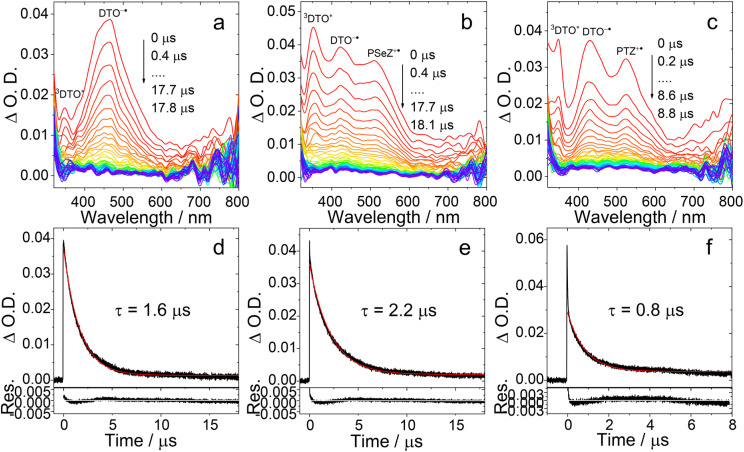
Nanosecond transient absorption spectra of (a) PTZ-DTO, (b) PSeZ-DTO and (c) DPTZ-DTO. Decay curves of (d) PTZ-DTO, (e) PSeZ-DTO at 460 nm and (f) DPTZ-DTO at 530 nm after pulsed laser excitation at 355 nm. *c* = 2.0 × 10^−5^ M in deaerated TOL, 25 °C.

We then checked the slower dynamics by measuring the ns-TA spectra of the compounds in deaerated TOL. For PTZ-DTO, we observed a broad positive absorption band centered at 465 nm and extending in the 400–600 nm spectral range (Fig. 6a). Based on the spectroelectrochemical results (Fig. S16), this band is assigned to a mixture of ^3^LE and ^3^CS states. This result is consistent with the DFT theoretical calculations. The lifetime of the transient species was determined as 1.6 μs (Fig. 6d), which is close to the delayed fluorescence lifetime of this compound (1.4 μs). Moreover, the lifetimes determined by monitoring the decay traces at 350 nm and 465 nm are same, indicating that the ^3^LE and ^3^CS states are in equilibrium. This observation is different from a previous study, showing only the ^3^LE state for some TADF emitters when using ns-TA spectroscopy.^42^

In case of PSeZ-DTO (Fig. 6b), we noticed two distinct absorption peaks, centered at 420 nm and 510 nm. Based on the spectroelectrochemistry outcomes (Fig. S16), we assign the band centered at 420 nm to the DTO^−^˙ radical anion, and that at 510 nm to the PSeZ^+^˙ radical cation. Another absorption band was also observed, centered at 350 nm, which is assigned to the ^3^LE state. The lifetime of PSeZ-DTO was determined as 2.2 μs (Fig. 6b). Interestingly, the transient lifetime of PSeZ-DTO resulted longer than that of PTZ-DTO, which is against the previous reports, claiming that RISC can be enhanced by incorporation of Se atom in the molecule.^58–61^ For DPTZ-DTO (Fig. 6c), we also observed an absorption band centered at 420 nm, attributed to the anion, and the band centered at 510 nm, attributed to the cation, besides an absorption band centered at 350 nm attributed to ^3^DTO* (Fig. S18). The excited state lifetime was determined as 0.8 μs, which is consistent with the delayed fluorescence lifetime (0.8 μs). Thus, for all compounds we observed both the ^3^LE and ^3^CS states in the ns-TA spectrum, which provides an experimental basis for the spin-vibronic coupling mechanism of TADF.^25,50^

**Fig. 7:**
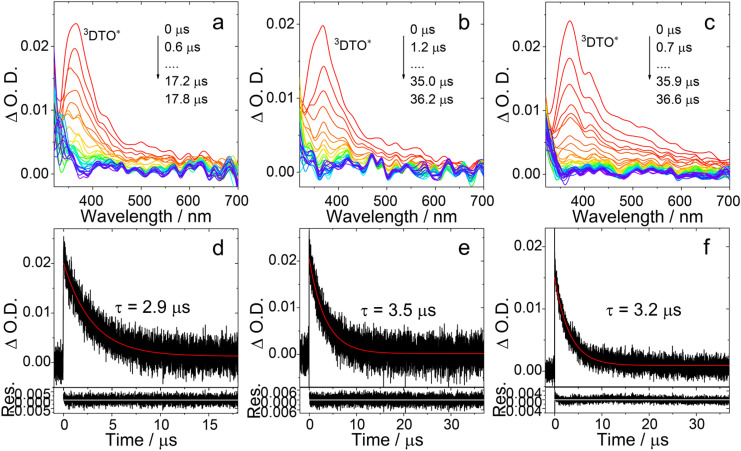
Nanosecond transient absorption spectra of (a) PTZ-O-DTO, (b) PTZ-O2-DTO and (c) DPTZ-O-DTO. Decay traces of (d) PTZ-O-DTO, (e) PTZ-O2-DTO and (f) DPTZ-O-DTO at 370 nm after pulsed laser excitation at 355 nm. *c* = 2.0 × 10^−5^ M in deaerated TOL, 25 °C.

We also measured the ns-TA spectra of the three compounds in HEX (Fig. S17). For PTZ-DTO, we observed a positive absorption band centered at 465 nm, narrower than that observed in TOL. For PSeZ-DTO, the absorption band centered at 350 nm, which is attributed to the ^3^LE state, is more significant. This result demonstrates that in HEX the CS state of PSeZ-DTO has higher energy than the ^3^LE state, and that the ^3^CS state is not significantly populated, also suppressing TADF. For DPTZ-DTO, we still observed a broad positive band centered at 465 nm and ranging between 400 and 600 nm, assigned to the mixture of ^3^LE and ^3^CS states. For this compound, the ^3^LE and ^3^CS state energy levels are close to each other both in HEX and TOL solutions, making TADF efficient in both solvents (Fig. S17). Finally, we measured the ns-TA spectra of PTZ-O-DTO, PTZ-O2-DTO and DPTZ-O-DTO (Fig. 7), observing similar spectra. For all of them we notice an absorption band centered at 360 nm, attributed to the ^3^LE state, while the ^3^CS state is no longer observed. These results infer that the energy of the ^3^CS state is higher than that of the ^3^LE state, in agreement with the electrochemical studies and DFT calculation (Table 4). Moreover, we did not observe the rise phase of the ns-TA spectra of the compounds, indicating that the formation of the long-lived species (^3^CS state and the ^3^LE state) is fast and occurs on a shorter time scale than the instrument response function (IRF) of the ns-TA spectrometer (*ca.* 10 ns).

Based on the ns-TA spectral data and the delayed fluorescence quantum yields of the compounds, we calculated the RISC rate constants according to a literature method.^41^ For PTZ-DTO, the rate constants of the RISC was calculated as *k*_RISC_ = 6.1 × 10^6^ s^−1^, for PSeZ-DTO it resulted *k*_RISC_ = 4.9 × 10^6^ s^−1^, and a value of *k*_RISC_ = 3.6 × 10^6^ s^−1^ was obtained for DPTZ-DTO. It is clear that, at least in our case, incorporation of heavy atom Se in the molecular structure does not enhance the reverse ISC process, although in some Se-substituted TADF emitters^58^ an heavy atom effect on the RISC process was indeed observed (with replace of S atom with Se atom, the *k*_RISC_ of the TADF emitters increased from 1.2 × 10^4^ s^−1^ to *k*_RISC_ = 2.8 × 10^5^ s^−1^). For the representative TADF compound 4Cz-IPN,^84^*k*_RISC_ resulted 6.8 × 10^6^ s^−1^, while for the brominated or iodinated 4Cz-IPN, the RISC rate constant was *k*_RISC_ = 7 × 10^7^ s^−1^. Therefore, in this case the so-called heavy atom effect for the RISC was observed. However, it should be pointed out that other factors, such as the planarity of the chromophores, also play a role in determining the RISC rate constants.^35,41,85^ Thus, one should be careful in the interpretation of the photophysical properties of the electron donor–acceptor TADF emitters.

### Time-resolved electron paramagnetic resonance (TREPR) spectra

**Fig. 8:**
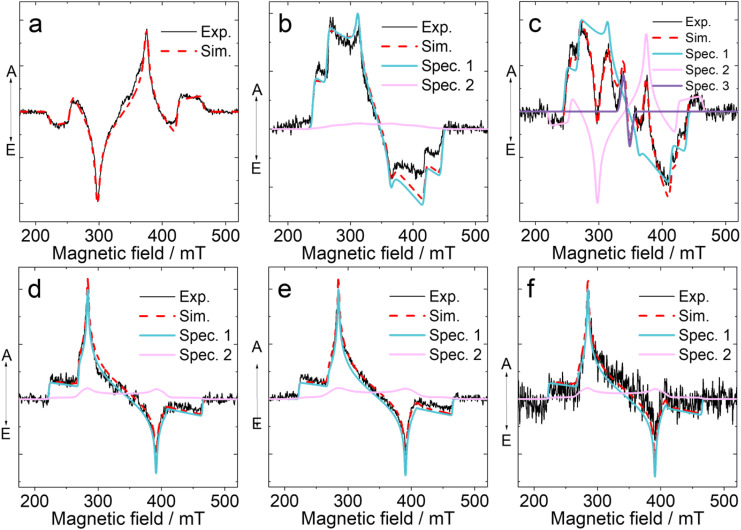
Experimental (black) and simulated (red) TREPR spectra of (a) PTZ-DTO, (b) PSeZ-DTO, (c) DPTZ-DTO, (d) PTZ-O-DTO, (e) PTZ-O2-DTO and (f) DPTZ-O-DTO with parameters given in Table 5. The experimental spectra are shown for a 500 ns delay after the laser flash with an integration window of 100 ns. The laser excitation wavelength is 355 nm, *c* = 5.0 × 10^−4^ M in solvents of TOL/2MeTHF (3/1, v/v). Blue and purple color represent the decomposition of the experimental spectrum into two spectra with similar *D*, *E* parameters but multiplet and net polarizations, respectively. Three different triplet states were used in the simulation spectrum of DPTZ-DTO (see Table 5 for details). The spin-polarised patterns are characterised by absorptive (A) and emissive (E) features: positive features are in enhanced absorption (A) and negative features are in emission (E).

We then investigated the transient paramagnetic species involved in the TADF process using pulsed laser excited TREPR (Fig. 8). In the absence of an external magnetic field, the splitting of triplets is described by the Zero Field Splitting (ZFS) *D* and *E* parameters, the main contribution to which is given by the dipole–dipole interaction between the two unpaired electrons of the triplet state. The ^3^CS state and the ^3^LE state can be easily discriminated with this technique, based on their distinctly different ZFS *D* parameters.^22,48,49,74^^3^LE state usually have a larger ZFS *D* parameter than the ^3^CS state because of the smaller average distance between the electrons for the ^3^LE state. It is known that during photoexcitation of chromophores, the sublevels of the photoexcited triplet states are populated selectively, *i.e.* an electron spin polarization (ESP) is resulted and the ESP phase pattern of the TREPR spectra differs depending on which sublevels are overpopulated. Moreover, the ISC mechanism leading to the population of the ^3^LE state can be also elucidated, based on the ESP of the triplet excited state TREPR spectra,^50,88–92^ although only the RP-ISC mechanism gives a unique ESP phase pattern of the triplet state.^22^

The spectra of PTZ-DTO were acquired in a frozen solution with mixed solvent TOL/2-methylTHF (3 : 1, v/v) (Fig. 8a). The obtained spectrum is typical for a triplet state of powder sample (randomly oriented molecules in the magnetic field), however, the ESP phase pattern, which is (e, a, e, a, e, a) (e: emission; a: enhanced absorption), corresponds to the overpopulation of the Ty sublevels of the triplet state and is different from that of an ordinary triplet state formed by the spin–orbit coupling (SOC) ISC mechanism, which usually give an ESP phase pattern (e, e, e, a, a, a) or (a, a, a, e, e, e). This result allows us to assume that the triplet state of PTZ-DTO may be produced by a mechanism other than SOC-ISC, as for instance, the SOCT-ISC,^43^ considering the orthogonal geometry of the molecular structures.^93^ A RP-ISC mechanism can be excluded based on the ESP of the triplet state.^48,94,95^ The ZFS of |*D*| and |*E*| were determined as 3400 MHz (121.3 mT) and 425 MHz (15.2 mT), respectively, by simulation of the spectrum (the sign of *D* is not defined, but *E* and *D* values are of the opposite sign, Table 5).

**Table 5:** Zero field splitting parameters (*D* and *E*) and relative population ratios p_*x*_, p_*y*_, p_*z*_ of the zero field spin states of the compounds^a^

Compounds	|*D*| (MHz); |*E*| (MHz)^b^	p_*x*_ : p_*y*_ : p_*z*_
PTZ-DTO	[3400; 425]	0.00 : 1.00 : 0.00
PSeZ-DTO	[2900; 480], spec. 1	0.00 : 0.00 : 1.00
	[2900; 480], spec. 2	Net-polarization
DPTZ-DTO	[2700; 450], spec. 1	0.00 : 0.17 : 0.83
	[3400; 425], spec. 2	0.00 : 1.00 : 0.00
	SCRP, Dz = −200, spec. 3	Triplet precursor
PTZ-O-DTO	[3400; 142], spec. 1	0.26 : 0.00 : 0.74
	[3400; 142], spec. 2	Net-polarization
PTZ-O2-DTO	[3400; 162], spec. 1	0.31 : 0.00 : 0.69
	[3400; 162], spec. 2	Net-polarization
DPTZ-O-DTO	[3400; 162], spec. 1	0.33 : 0.00 : 0.67
	[3400; 162], spec. 2	Net-polarization

a Obtained from simulations of the triplet state TREPR spectra of the indicated compounds in TOL/2MeTHF (1/3) at 80 K. *g*-Factor is 2.0030 ± 0.0005 for all spectra. Gaussian line width (peak-to-peak) is 20 mT for spectra with net polarization and it is 2 mT for all other spectra. The error of the ZFS parameters does not exceed 5%.

b It is impossible to determine the sign of the parameters from the analysis of the EPR spectra, therefore the absolute values of the parameter *D* and the sign of *E* are given from the condition (−1/3*D* ≤ *E* ≤ 0).^96,97^

We also measured the TREPR spectrum of PTZ-DTO in a solvent with slightly higher polarity (Fig. S19), observing a different polarization pattern. The spectrum is described as the sum of the spectra of the localized ^3^LE state, with a ZFS *D* parameter of 2900 MHz and the spectrum of the SCRP. To describe the experimentally observed asymmetry of the triplet spectrum, we added a signal from the net polarization of the ^3^LE state (second component), although the asymmetry may have a different nature. A satisfactory simulation of the spectrum requires three components (Table S1), two species are attributed to the ^3^LE state, with a ZFS *D* parameter of 2900 MHz and with different polarization types: multiplet and net polarizations. The third component is assigned as a SCRP from a triplet precursor,^98^ the simulation gives a *D*_*Z*_ magnitude of −200 MHz assuming dipole interaction in a SCRP, but the contribution from the exchange interaction cannot be excluded. The CS state in the TREPR of PTZ-DTO in DCM/2MeTHF is in agreement with observations made in the fs-TA spectra.

For PSeZ-DTO (Fig. 8b), the ESP phase pattern of the triplet state TREPR spectrum is (a, a, a, e, e, e), which is typical for a triplet state formed by the normal SOC-ISC.^88,94^ The spectral simulation indicates that the ZFS *D* parameter is 2900 MHz, and the population ratio is p_*x*_ : p_*y*_ : p_*z*_ = 0.00 : 0.00 : 1.00. A similar narrow signal was also observed for the DPTZ-DTO compound, in contrast to the PTZ-DTO compound. A narrow signal was observed for DPTZ-DTO both for the frozen of DCM/2MeTHF and TOL/2MeTHF. The spectrum of the triad DPTZ-DTO is more complicated than that of PTZ-DTO (Fig. 8c). A satisfactory simulation requires three components, two of them, having the ZFS *D* parameters of 2700 MHz and 3400 MHz respectively, are assigned as two different triplet states, showing different population ratios of three sublevels. The spectrum with *D* parameter of 3400 MHz completely coincides with spectrum of PTZ-DTO (Table 5). Increasing the delocalization in this case leads to a decrease in the parameter *D*. The third component is as already noted a SCRP, with electron spin dipolar interaction of −200 MHz (Table 5), and it appears as the precursor of the T_0_ substrate. Since DPTZ-DTO shows significant TADF, we propose that this is another example of interconversion between the ^3^LE state and the ^3^CS state, *i.e.*, this is again an experimental evidence for the spin-vibronic coupling model of the TADF mechanism.

The effect of oxidation of the PTZ moiety on the photophysics of the TADF emitters was also studied with TREPR spectroscopy (Fig. 8d). The ESP of the triplet state TREPR spectrum of PTZ-O-DTO is (a, a, a, e, e, e), which is the typical ESP phase pattern of a triplet state produced by SOC-ISC.^88,94^ The spectral simulation gives a ZFS *D* parameter of 3400 MHz, which is literally the same of that of PTZ-DTO (Table 5). However, the population ratio of the triplet state is p_*x*_ : p_*y*_ : p_*z*_ = 0.26 : 0.00 : 0:74, which is different from that of PTZ-DTO.

These results indicate that there is an equilibrium between ^3^LE state and ^3^CS state, supporting the spin-vibronic mechanism for TADF. Other TADF molecules have been investigated by TREPR spectroscopy in previous studies, showing a ^3^CS state with a *D* value from 430 to 1440 MHz.^43,49,51,67^

Thus, we assume that in PTZ-DTO, the ISC mechanism is probably SOCT-ISC, whereas for PTZ-O-DTO, it is SOC-ISC. Note that the triplet spectrum of PTZ-O-DTO has no symmetric form which may be a result of the triplet net polarization. Considering the contribution from net polarization of this triplet gives a satisfactory description of the experimental spectrum (Table 5). Further evidence for this conclusion comes from PTZ-O2-DTO (Fig. 8e). For this compound, a TREPR spectrum with (a, a, a, e, e, e) electron spin polarization phase pattern was observed, which is attributed to a ^3^LE state. The simulation of the spectrum gives a ZFS *D* parameter of 3400 MHz, similar to that of PTZ-O-DTO (*D* = 3400 MHz) and PTZ-DTO (*D* = 3400 MHz) (Table 5).

Similar results were obtained for DPTZ-O-DTO (Fig. 8f and Table 5). Thus, we propose that for all the oxidized compounds, the T_1_ state (^3^LE) is confined on the DTO unit. The above TREPR spectral studies demonstrated a few critical aspects of the studied electron D–A compounds. First, the ISC mechanism of these TADF emitters most likely is SOCT-ISC, not the normal SOC-ISC. Second, the CS state (long-lived one) is formed *via* the ^3^LE state, thus verifying the spin-vibronic coupling model. Theoretical computations can reproduce the ZFS *D* magnitudes to some extents (Table S2). We also calculated the ZFS principal axis (Fig. S26). However, we did not make a detailed correlation between the theoretical calculated population ratios of the three sublevels of the T_1_ state of the compounds with the TREPR spectral results.

### Theory calculations

**Fig. 9:**
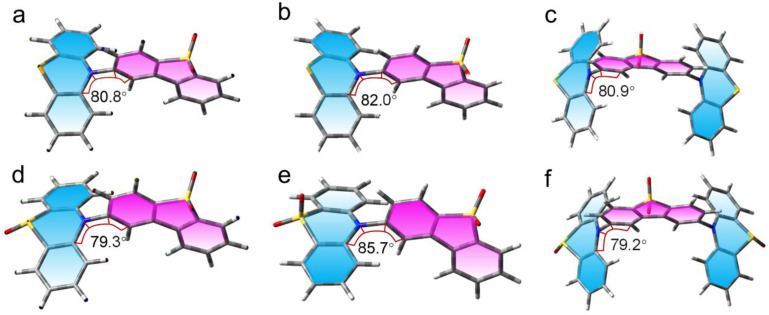
Optimized ground state geometry of (a) PTZ-DTO, (b) PSeZ-DTO, (c) DPTZ-DTO, (d) PTZ-O-DTO, (e) PTZ-O2-DTO and (f) DPTZ-O-DTO in gas phase, the blue and pink sheets show the planes of the donor and the acceptor, respectively, isovalue = 0.02. The selected dihedral angles between the electron donor and acceptor are presented. Calculations were performed with DFT theory at the B3LYP/6-31G(d) level with Gaussian 16.

**Fig. 10:**
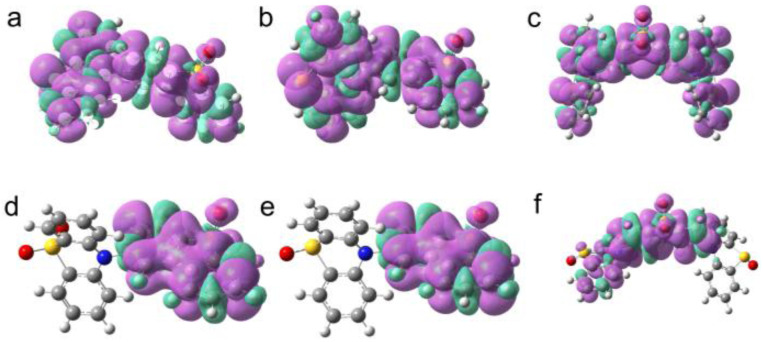
Spin density surfaces of the dyads in the T_1_ state of (a) PTZ-DTO, (b) PSeZ-DTO, (c) DPTZ-DTO, (d) PTZ-O-DTO, (e) PTZ-O2-DTO and (f) DPTZ-O-DTO in TOL, isovalue = 0.02. Calculations were performed with DFT at the UB3LYP/6-31G(d) level with Gaussian 16.

Finally, we performed a theoretical analysis to substantiate and further interpret our data. The ground state geometry of the compounds has been optimized at DFT-B3LYP/6-31G(d) level (Fig. 9). The dihedral angle between DTO and PTZ resulted *ca.* 80°, which is close to a vertical configuration. PTZ presents a puckered conformation at the ground state, while DTO adopts a planar geometry. The triplet state spin density of the compounds with PTZ unit oxidized in TOL were also analyzed (Fig. 10). For PTZ-DTO, PSeZ-DTO and DPTZ-DTO, the triplet state density is distributed on PTZ and DTO. The ns-TA spectra indicated that the T_1_ state is not a pure ^3^DTO* state or a pure ^3^CS state, but an equilibrium between ^3^LE/^3^CS is established. For the oxidized compounds PTZ-O-DTO and PTZ-O2-DTO (Fig. 10), the triplet state density is confined on the DTO moiety, confirming that the T_1_ state is a ^3^LE state, as experimentally observed. Other functional and basis sets were tested, but generally the same results were observed (Fig. S25).

In the polar solvent ACN, the spin density surface of the T_1_ state of the dyads with the PTZ part oxidized is localized on the DTO moieties (Fig. S24). These results are in agreement with ns-TA spectroscopy (Fig. 7). For the dyads and the triad where the PTZ unit is oxidized, the T_1_ state is also a ^3^LE state, in agreement with the TREPR results. For DPTZ-O-DTO the triplet state spin density is shown to be a mixture of ^3^LE and ^3^CS states, whereas only ^3^LE state is observed in ns-TA and TREPR spectra (Fig. 7 and 8).

**Scheme 2:**
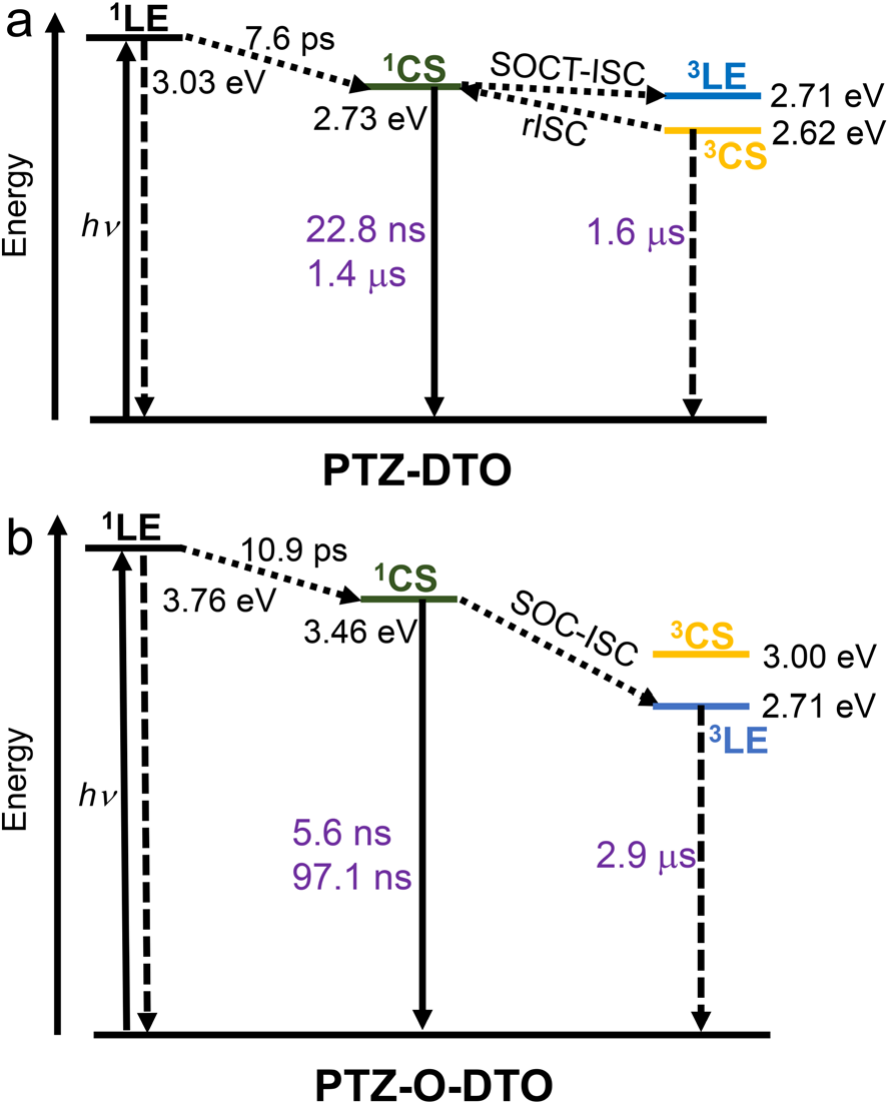
Photophysical process of (a) PTZ-DTO and (b) PTZ-O-DTO in TOL. The ^1^LE and ^3^CS state energy is calculated by TDDFT at the B3LYP/6-31G(d) level using Gaussian 16. The ^1^CS state energy is obtained by electrochemical calculations (the energy levels of the ^1^CS state obtained from TDDFT are as follows: PTZ-DTO: 2.62 eV; PTZ-O-DTO: 3.42 eV) the ^3^LE state energy is estimated by the vibrational 00 T_1_ → S_0_ transition based on phosphorescence spectra.

The photophysical processes of PTZ-DTO and PTZ-O-DTO are summarized in Scheme 2. Upon photoexcitation, the ^1^LE state is populated for both compounds, which undergoes a rapid charge separation to give the ^1^CS state. For PTZ-DTO, the ^1^CS, ^3^CS and ^3^LE states have similar energies, and the ^3^LE and ^3^CS states are observed simultaneously in the ns-TA spectra, justifying the observed TADF. For the compounds with the oxidized PTZ unit, the energy of the CS state increases, while no change in energy occurs for the ^3^LE state energy. These compounds thus present a large energy gap between the ^3^LE state and the CS state, and consequently only the ^3^LE state is observed in the ns-TA spectra. In this case the spin-vibronic coupling between ^3^LE and ^3^CS is weak and it is unlikely to observe significant TADF.

## Conclusions

In summary, we studied the electronic excited states dynamics and electron spin dynamics of a series representative thermally activated delayed fluorescence (TADF) emitters based on phenothiazine–dibenzothiophene-*S*,*S*-dioxide (PTZ-DTO) dyads using steady state and femtosecond/nanosecond transient absorption (fs/ns-TA) spectroscopy, as well as pulsed laser-excited time-resolved electron paramagnetic resonance (TREPR) spectroscopy. The molecular structures were systematically varied by attaching an extra electron donor (PTZ) to the electron acceptor (DTO), using Se atom to replace the S atom or by oxidation of the PTZ unit to sulfoxide and sulfone. Fs-TA spectroscopy shows that charge separation (CS) occurs readily (always in <20 ps) in all the dyads where the donor is not oxidized, while charge recombination (CR) is quite slow (>5 ns) in toluene, but is accelerated in polar solvents. Ns-TA spectroscopy demonstrated the coexistence of the ^3^CS and ^3^LE states for the non-oxidized dyads. Oxidation of the PTZ unit increases the energy of the CS state and as a result, only the ^3^LE (localized on the DTO moiety) state is observed. In the TREPR spectra we observed an electron spin polarization (ESP) phase pattern (e, a, e, a, e, a) for the PTZ-DTO dyad, suggesting triplet formation through the spin–orbit charge transfer intersystem crossing (SOCT-ISC) mechanism, rather than the spin–orbit coupling ISC (SOC-ISC) mechanism. However, upon Se substitution, SOC-ISC becomes dominant. In solvents with slightly higher polarity, a spin-correlated radical pair (SCRP) was also observed (dipolar interaction is −200 MHz), supporting the spin-vibronic coupling mechanism for TADF. These studies are useful for an in-depth understanding of the photophysics of the electron donor–acceptor TADF emitters and for the design of new light emitting materials for OLED applications. A key finding is that the strategy proposed in previous studies, introducing heavy atoms to reduce the efficiency roll-off in OLED devices, is not a universal rule. This study found that in our compounds the introduction of heavy atoms does not perturb the decay lifetimes of delayed fluorescence and triplet states. This result has significant implication for molecular structure design of electron donor–acceptor dyads as OLED emitters. When designing TADF molecules, the impact of structural modifications on the CS state energy level should also be evaluated. Moreover, deep understanding the factors dictating the triplet state lifetime of TADF emitters is important for device performance, because a long-lived triplet state may lead to efficiency roll-off of the OLED devices.

## Author contributions

Y. P. and A. A. S. contributed equally to this work. J. Z. conceived the work, acquired the funding, directed the data analysis and the manuscript writing; Y. P. synthesized the compounds, measured and analyzed steady-state optical spectra, electrochemistry, nanosecond transient absorption spectra, performed parts of the data analysis, and modified the manuscript; A. A. S. and V. K. V. measured the TREPR spectra and analyzed the data; G. S., L. B. and M. D. D. did the femtosecond transient absorption studies and analyzed the data. X. C., Y. L. and Y. H. revised parts of the manuscript; X. L. and H. G. performed the theoretical calculations and analyzed the data; all authors contributed to the writing of the manuscript.

## Conflicts of interest

There are no conflicts to declare.

## Supplementary Material

SC-016-D5SC03644E-s001

## Data Availability

All relevant data are presented in the main text and/or SI. Supplementary information: the molecular structural characterization date, UV-Vis absorption and fluorescence spectra, electrochemical studies, nanosecond and femtosecond transient absorption spectra, TREPR spectra and theoretical computation information. See DOI: https://doi.org/10.1039/d5sc03644e.
