# Supplementary material for: Study of the thermally-activated delayed fluorescence (TADF) mechanism of phenothiazine–dibenzothiophene-S,S-dioxide electron donor–acceptor dyads using steady-state and time-resolved optical and electron paramagnetic resonance spectroscopies
Source: Chem Sci. 2025 Sep 24;16(42):19737–51. doi: 10.1039/d5sc03644e (PMC12487830; doi:10.1039/d5sc03644e)
Supplement: SC-016-D5SC03644E-s001 [file SC-016-D5SC03644E-s001.pdf]

Supplementary Information (SI) for Chemical Science.
This journal is © The Royal Society of Chemistry 2025

Electronic Supplementary Information for:

Study of the Thermally-Activated Delayed Fluorescence (TADF) Mechanism of Phenothiazine-Dibenzothiophene-*S*,*S*-dioxide Electron Donor–Acceptor Dyads Using Steady-State and Time-Resolved Optical and Electron Paramagnetic Resonance Spectroscopies

Yuying Pei,[a†] Andrey A. Sukhanov,[c†] Xi Chen,[a] Greta Sambucari,[d] Laura Bussotti,[d] Xin Liu,[e] Jianzhang Zhao,[*,a] Yanqin Li,[*,b] Yanping Huo,[*,f] Violeta K. Voronkova,[*,c] Huimin Guo, [*, e] and Mariangela Di Donato,[*,d,g]

[a] State Key Laboratory of Fine Chemicals, Frontier Science Center for Smart Materials, School of Chemical Engineering, Dalian University of Technology, Dalian 116024, P. R. China. E-mail: zhaojzh@dlut.edu.cn

[b] School of Chemistry, Dalian University of Technology, Dalian 116024, P. R. China. E-mail: liyanqin@dlut.edu.cn

[c] Zavoisky Physical-Technical Institute, FRC Kazan Scientific Center of RAS, Kazan 420029, Russia. E-mail: vio18@kfti.knc.ru

[d] LENS (European Laboratory for Non-Linear Spectroscopy), Via N. Carrara 1, 50019, Sesto Fiorentino (FI), Italy. E-mail: didonato@lens.unifi.it

[e] School of Chemistry, Dalian Key Laboratory of Intelligent Chemistry, Dalian University of Technology, Dalian 116024, P. R. China. *Email: guohm@dlut.edu.cn

[f] School of Chemical Engineering and Light Industry, Guangdong University of Technology, Guangzhou 510006, P. R. China; E-mail: yphuo@gdut.edu.cn

[g] ICCOM-CNR, via Madonna del Piano 10-12, 50019, Sesto Fiorentino (FI), Italy

†These authors contributed equally to this work.

Index

1. General Experimental Information and Synthesis of the Compounds....S3

2. NMR and HRMS Spectra Data....S6

3. Fluorescence Spectra....S10

4. Fluorescence Lifetime Spectra....S11

5. Femtosecond Transient Absorption Spectra....S13

6. Spectroelectrochemistry....S14

7. Nanosecond Transient Absorption Spectra····S15

8. Time–Resolved Electron Paramagnetic Resonance Spectra ····S16

9. DFT Calculations····S18

10. References····S21

1. General Experimental Information and Synthesis of the Compounds.

All of the chemicals used in the synthesis are analytically pure and were used as received. Solvents were dried prior to use. UV/vis absorption spectra were recorded on a UV/2550 spectrophotometer (Shimadzu Ltd., Japan). Fluorescence emission spectra were recorded on a FS5 spectrofluorometer (Edinburgh Instruments, U.K.). Luminescence lifetimes were measured on OB920 luminescence lifetime spectrometer (Edinburgh Instruments, U.K.). Fluorescence quantum yields (Φ_F) were recorded with an absolute PL quantum yield spectrometer (Quantaurus-QY plus C13534-11, Hamamatsu Ltd., Japan).

1.1. Compound PTZ-O-DTO and **PTZ-O2-DTO**. Compounds **PTZ-O-DTO** and **PTZ-O2-DTO** were synthesized following the reported method.[1] **PTZ-DTO** (123.9 mg, 0.30 mmol) was dissolved in acetic acid (18.0 mL) under air atmosphere, then 30% H_2O_2 (0.40 mL, 0.40 mmol) was added. The mixture was stirred at 50 °C for 2 h. After the reaction was completed, the reaction mixture was cooled to room temperature and then poured into water (20 mL), then the mixture was extracted with dichloromethane (3 × 20 mL). The organic layer was dried over anhydrous Na_2SO_4 and the solvent was evaporated under reduced pressure. The crude product was purified by column chromatography (silica gel, Dichloromethane (DCM): Ethyl acetate (EA) = 2: 1, v: v). **PTZ-O-DTO** was obtained as white solid (50 mg, yield 39%). ^{1}H NMR (400 MHz, DMSO) δ 8.48 (s, 1H), 8.40 (d, J = 8.1 Hz, 1H), 8.29 (d, J = 7.6 Hz, 1H), 8.10 (d, J = 7.4 Hz, 3H), 7.83–7.79 (m, 1H), 7.76–7.71 (m, 2H), 7.58–7.54 (m, 2H), 7.37–7.33 (m, 2H), 6.82 (d, J = 8.5 Hz, 2H). ^{13}C NMR (125 MHz, DMSO): δ = 144.20, 137.83, 137.50, 137.17, 134.77, 134.67, 133.09, 132.96, 131.73, 131.57, 129.95, 125.51, 125.25, 123.56, 122.49, 122.43, 122.08, 117.01. ESI-HRMS $(C_{24}H_{15}NO_3S_2+Na)^+$: calcd *m/z* 452.0487; found *m/z* 452.0390. **PTZ-O2-DTO** was obtained as white solid (60 mg, yield 45%). ^{1}H NMR (400 MHz, $CDCl_3$) δ 8.22–8.17 (m, 3H), 7.92 (d, J = 7.5 Hz, 1H), 7.85 (s, 1H), 7.77 (d, J = 7.5 Hz, 1H), 7.72–7.61 (m, 3H), 7.47–7.44 (m, 2H), 7.34–7.30 (m, 2H), 6.68 (s, 2H). ESI-HRMS $(C_{24}H_{15}NO_4S_2+Na)^+$: calcd *m/z* 468.0436; found *m/z* 468.0339.

1.2. Compound DPTZ-O-DTO. Compound **DPTZ-O-DTO** was synthesized following the reported method.[1] The synthesis procedure is similar to that of **PTZ-O-DTO**. Yield: 52%. ^{1}H NMR (400 MHz, DMSO) δ 8.58 (s, 2H), 8.49 (d, J = 8.1 Hz, 2H), 8.05 (d, J = 7.5 Hz, 4H), 7.83 (d, J = 8.0 Hz, 2H), 7.56–7.52 (m, 4H), 7.33–7.29 (m, 4H), 6.80 (d, J = 8.5 Hz, 4H). ESI-HRMS $(C_{36}H_{22}N_2O_4S_3+Na)^+$: calcd *m/z* 665.0736; found *m/z* 665.0649.

1.3. Nanosecond transient absorption (ns-TA) spectroscopy. The ns-TA spectra were measured

on LP980 laser flash photolysis spectrometers (Edinburgh Instruments, U.K.). Samples were purged with N_2 for 15 min before measurements, and excited with a nanosecond pulsed laser (Surelite OPO Plus SL 1-10, Continuum Ltd., USA). The signal was digitized with a Tektronix TDS 3012B oscilloscope and the data was processed with L900 software.

1.4. Femtosecond transient absorption (fs-TA) spectroscopy. The fs-TA spectra were recorded using an experimental setup centered around a regenerative amplifier Ti:sapphire laser (Legend, Coherent), which was pumped by a Ti:sapphire oscillator (Micra, Coherent). The system produced 40 fs pulses at 800 nm with a repetition rate of 1 kHz and an average power of 3.2 W. For excitation, 350 nm pulses were generated through third harmonic generation of the signal beam. This signal beam was produced by directing a portion of the fundamental laser radiation to a commercial optical parametric amplifier (Topas, Light Conversion).

The probe beam was created by focusing a small fraction of the fundamental laser output onto a 3 mm thick CaF_2 crystal window, generating a white light continuum that spanned the spectral range from 375–750 nm. Temporal delays between pump and probe pulses were controlled using a motorized translation stage in the path of the 800 nm pulse used for white light generation. To account for intensity fluctuations, a portion of the white light continuum was split off as a reference beam through a separate optical path. Both pump and probe beams were focused onto the sample using a 75 mm spherical mirror, ensuring optimal spatial and spectral overlap at the sample position. The sample solution was contained in a 2 mm quartz cuvette, mounted on a movable stage to avoid photodegradation. All acquired data were processed using singular value decomposition and global analysis techniques implemented in the Glotaran software package.[2]

1.5. Time-resolved electron paramagnetic resonance (TREPR) spectra. The samples were prepared at a concentration of 5×10^{-4} M in a mixed solvent system of toluene (TOL)/(2-methyltetrahydrofuran, 2-MeTHF) = 1/3 (v/v) and DCM/2-MeTHF=1/3 (v/v). Time-resolved continuous-wave (CW) EPR spectra were performed on an X-band EPR Elexsys E-580 spectrometer (Bruker) equipped with the dielectric ring X-Band ER 4118X-MD5-W1 resonator at 80 K. The samples were excited at a wavelength of 355 nm, a pulse energy of 1 mJ and frequency of 100 Hz. The acquired EPR spectra were analyzed and simulated using the EasySpin toolbox implemented in MATLAB.[3]

1.6. Density functional theory (DFT) calculations. DFT calculations were carried out using the Gaussian 16 software package.[4] The ground state geometries were optimized using DFT at the

B3LYP[5]/6-31G(d)[6] and B3LYP/GENECP (LanL2DZ)[7] level. To determine the triplet excited-state energies, time-dependent DFT (TDDFT) calculations were performed at the B3LYP/6-31G(d) level, using the optimized ground-state geometries as the initial geometry. The solvent model in the computation is the CPCM model[8].

1.7. Electrochemical studies. The cyclic voltammograms were performed on a CHI610D electrochemical workstation (CHI instruments, Inc., Shanghai, China). Prior to measurements, the sample solutions were purged with N_2 for 15 min and N_2 atmosphere was maintained during the measurement. 0.10 M $Bu_4N[PF_6]$ was used as a supporting electrolyte; a platinum electrode as the counter electrode; glassy carbon electrode as the working electrode and the Ag/$AgNO_3$ (0.1 M in acetonitrile (ACN)) couple was used as the reference electrode. Ferrocenium/ferrocene (Fc^+/Fc) redox couple was used as an internal reference.

1.8. Singlet oxygen quantum yield (Φ_Δ). 1,3-Diphenylisobenzofuran (DPBF) was used as $^1O_2$ chemical trap. The sample solutions containing DPBF were irradiated, and the decay of DPBF absorbance at 414 nm was monitored over time. The value of Φ_Δ was obtained by the relative method by using Equation (1):[9]

$$\Phi_{\mathrm{sam}} = \Phi_{\mathrm{std}}\left(\frac{1-10^{-A_{\mathrm{std}}}}{1-10^{-A_{\mathrm{sam}}}}\right)\left(\frac{1-10^{-m_{\mathrm{std}}}}{1-10^{-m_{\mathrm{sam}}}}\right) \quad (1)$$

In the above equation, 'sam' and 'std' represent sample and standard, respectively. Φ, A, m and η represent the singlet oxygen quantum yield, the absorbance at excitation wavelength, the slope of the absorbance of DPBF over time, and the refractive index of the solvent used for measurement, respectively, $Ru(bpy)_3[PF_6]_2$ as standard (Φ_{std} = 0.57 in DCM). Optically matched solutions were used (the solutions of the sample and the standard have the same absorbance at the excitation wavelength). The obtained absorbance values were fitted with the illumination time to obtain the Φ_Δ value.

2. NMR and HRMS Spectra Data.

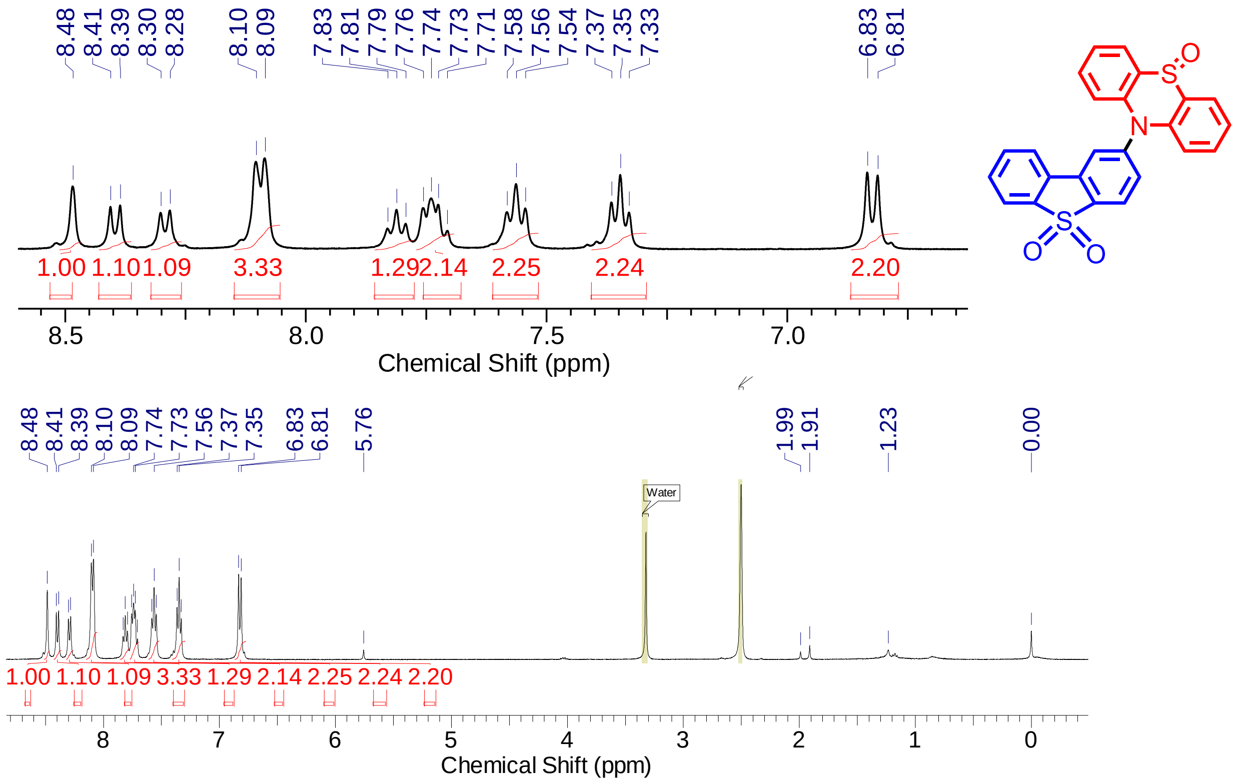

Fig. S1 ^{1}H NMR spectrum of compound **PTZ-O-DTO** (400 MHz, DMSO-d_6).

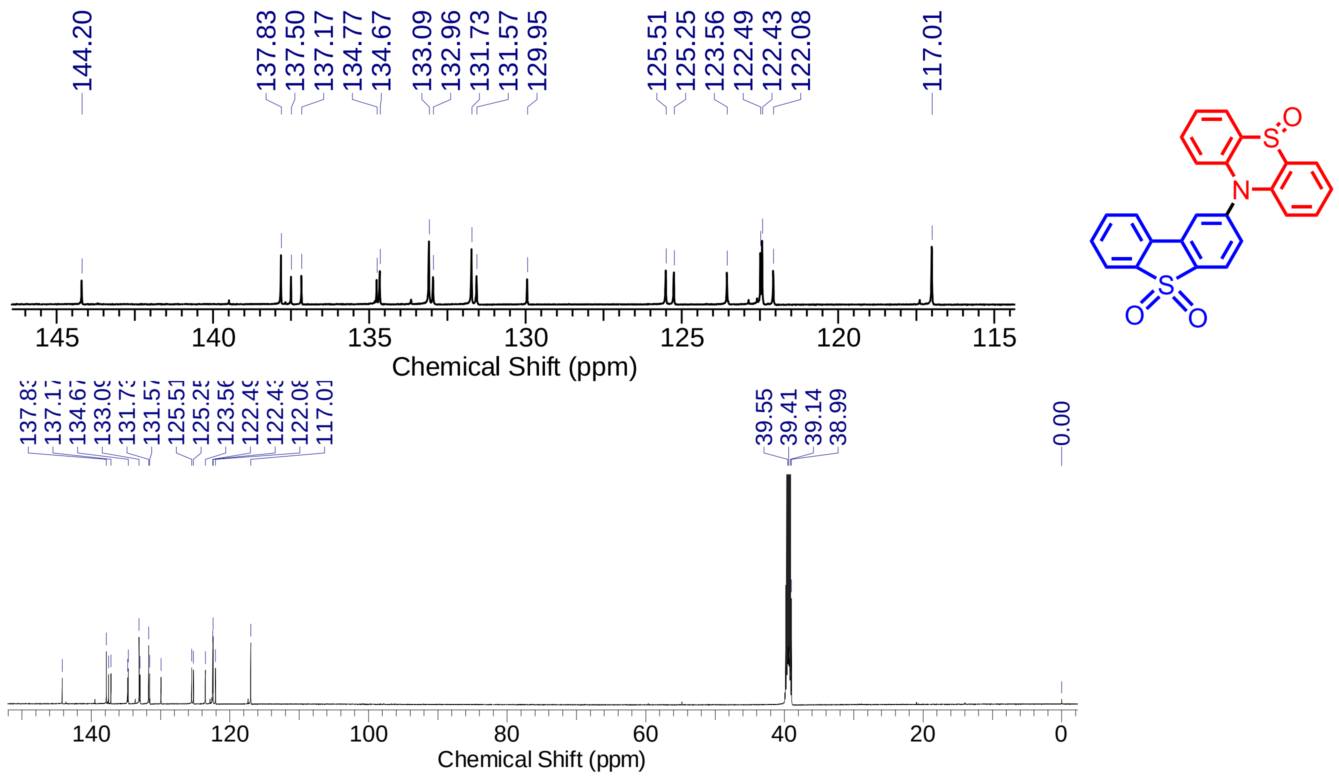

Fig. S2 ^{13}C NMR spectrum of compound **PTZ-O-DTO** (125 MHz, DMSO-d_6).

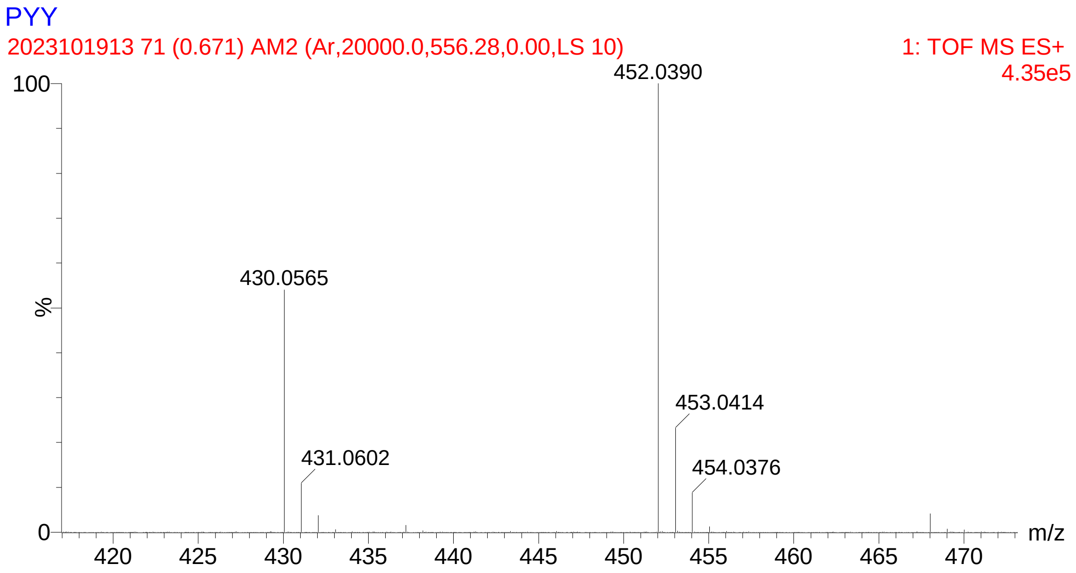

Fig. S3 ESI–HRMS spectrum of compound **PTZ-O-DTO**.

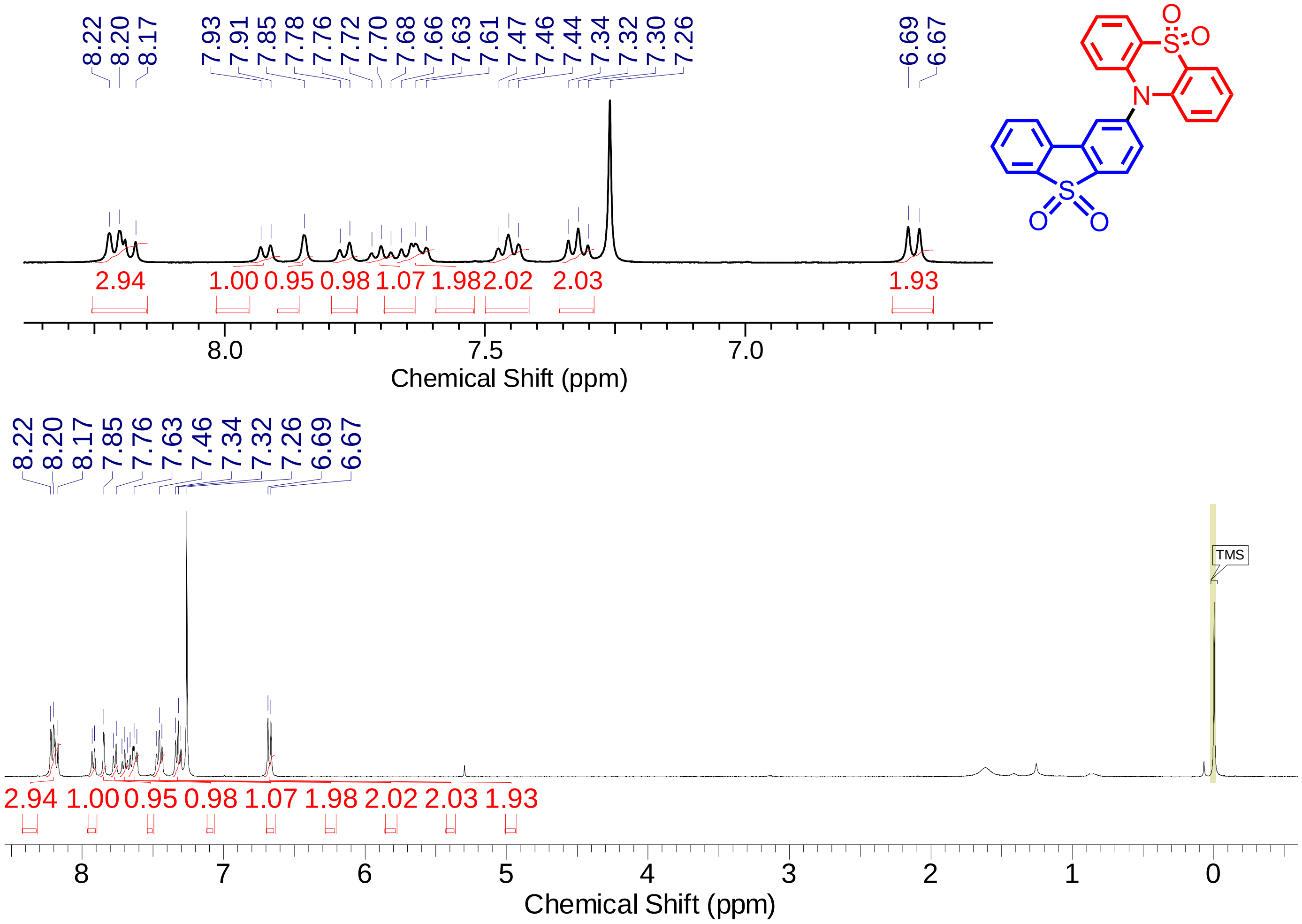

Fig. S4 ^{1}H NMR spectrum of compound **PTZ-O2-DTO** (400 MHz, $CDCl_3$).

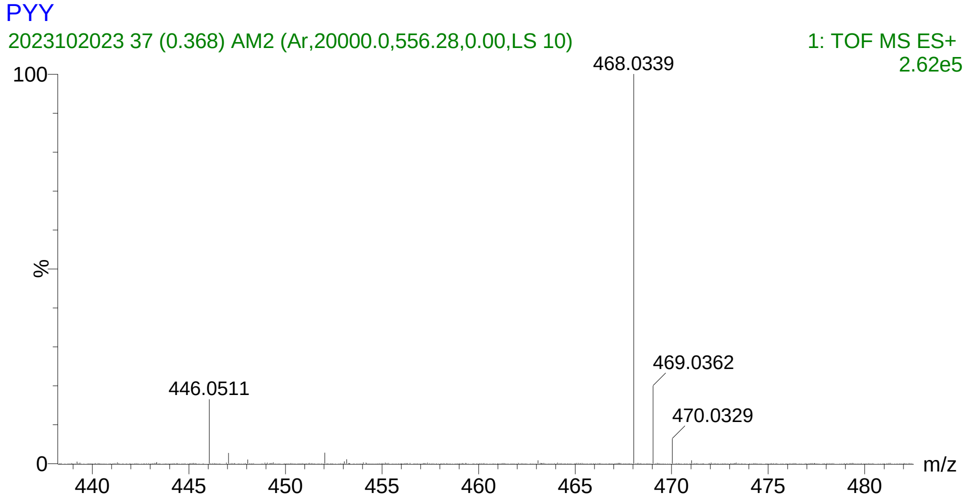

Fig. S5 ESI–HRMS spectrum of compound **PTZ-O2-DTO**.

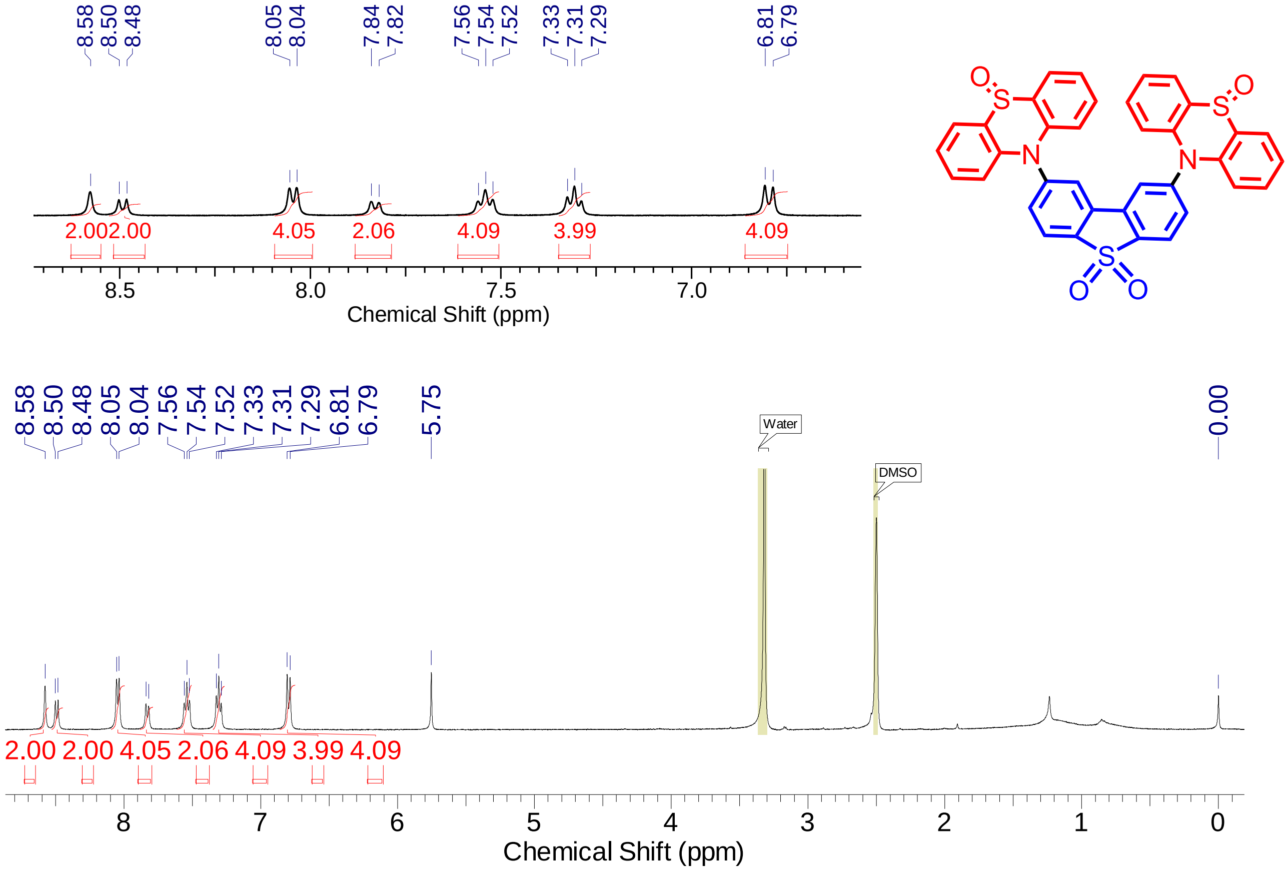

Fig. S6 ^{1}H NMR spectrum of compound **DPTZ-O-DTO** (400 MHz, $CDCl_3$).

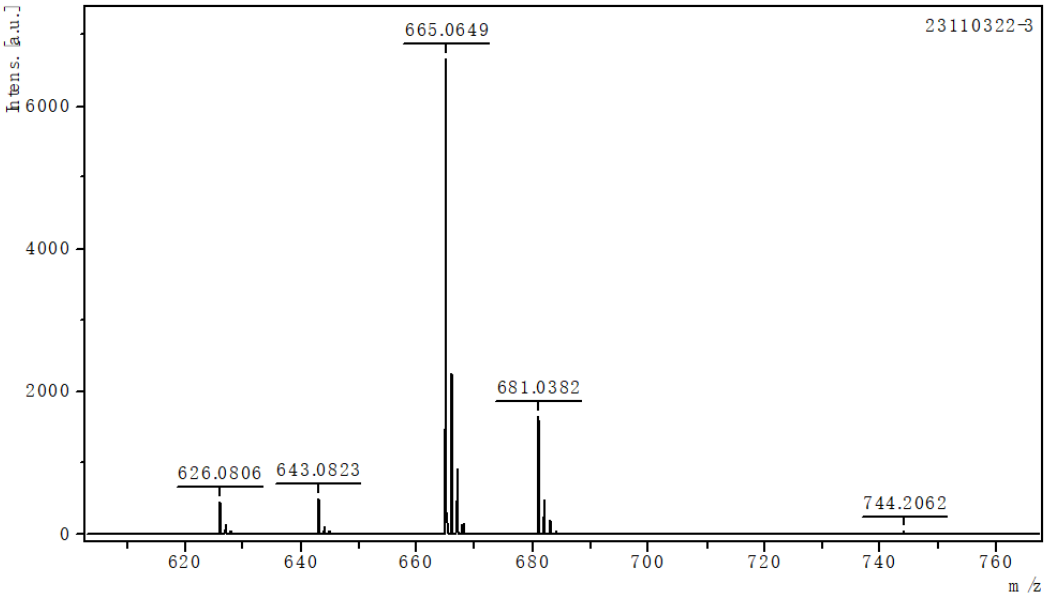

Fig. S7 MALDI–TOF–HRMS spectrum of compound **DPTZ-O-DTO**.

3. Fluorescence Spectra.

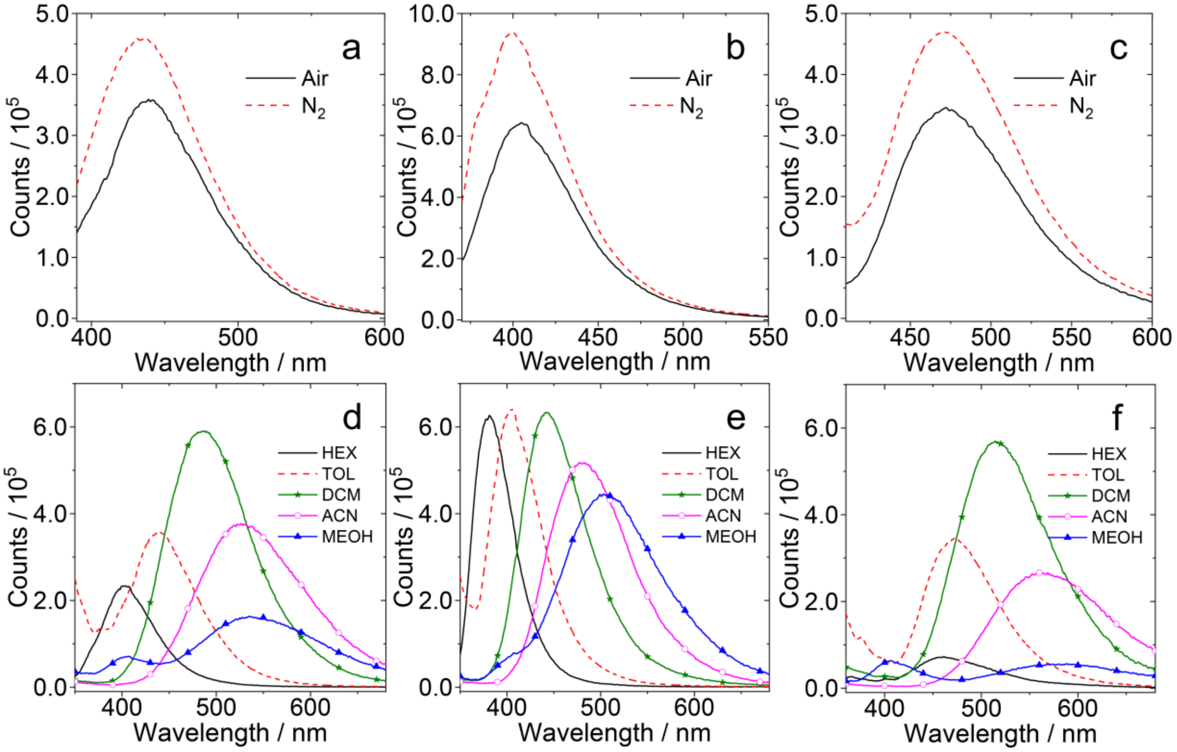

Fig. S8 Fluorescence spectra of (a) **PTZ-O-DTO**, (b) **PTZ-O2-DTO**, (c) **DPTZ-O-DTO** in TOL, (d) **PTZ-O-DTO**, (e) **PTZ-O2-DTO** and (f) **DPTZ-O-DTO** in aerated solution. $c = 1.0 \times 10^{-5}$ M, λ_{ex} = 320 nm, 25 °C.

4. Fluorescence Lifetime Spectra.

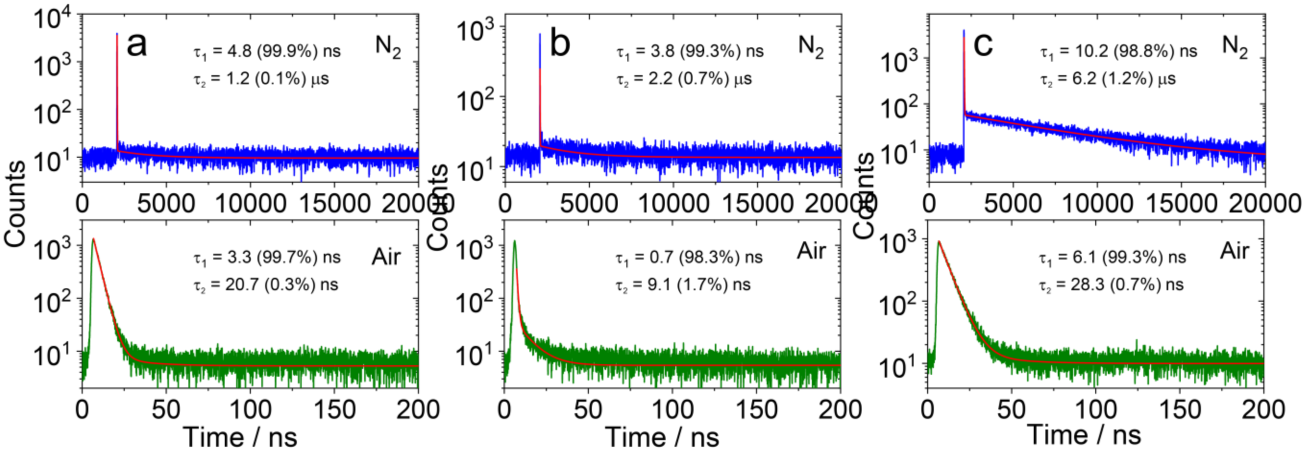

Fig. S9 Fluorescence decay traces of the (a) **PTZ-DTO** were monitored at 500 nm, (b) **PSeZ-DTO** were monitored at 490 nm and (c) **DPTZ-DTO** were monitored at 530 nm under different atmosphere (N_2, Air) in hexane (HEX). $c = 1.0 \times 10^{-5}$ M, λ_{ex} = 340 nm, 25 °C.

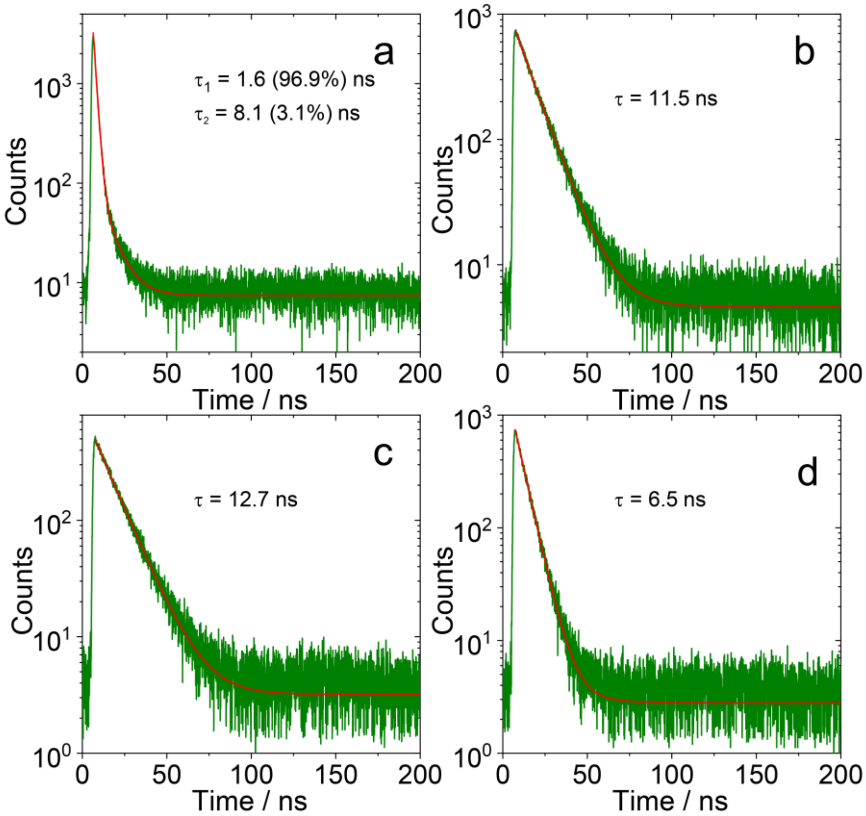

Fig. S10 Fluorescence decay traces of the **PTZ-O-DTO** were monitored under different atmosphere (N_2, Air): (a) HEX at 420 nm, (b) DCM at 480 nm, (c) ACN at 500 nm and (d) methanol (MeOH) at 530 nm, $c = 1.0 \times 10^{-5}$ M, λ_{ex} = 340 nm, 25 °C.

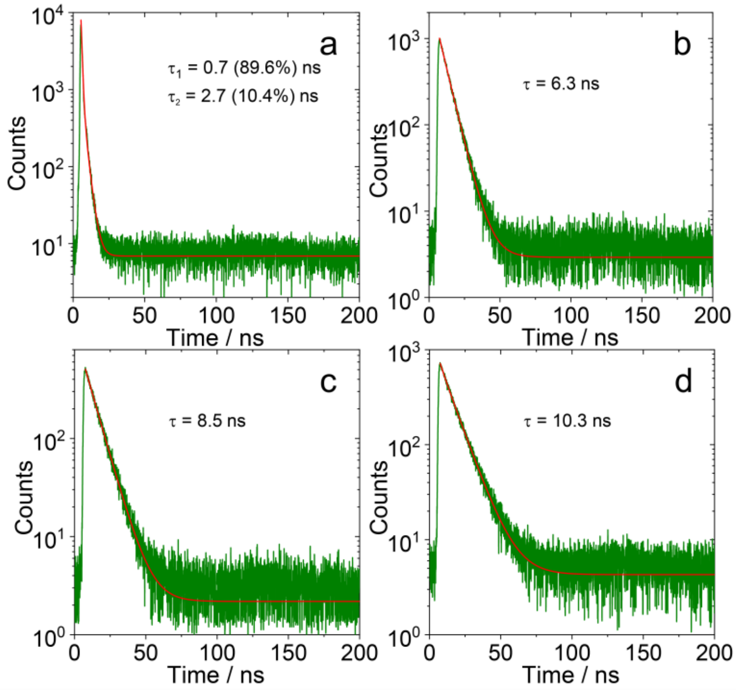

Fig. S11 Fluorescence decay traces of the **PTZ-O2-DTO** were monitored under different atmosphere (N_2, Air): (a) HEX at 360 nm, (b) DCM at 440 nm, (c) ACN at 480 nm and (d) MeOH at 500 nm, $c = 1.0 \times 10^{-5}$ M, λ_{ex} = 340 nm, 25 °C.

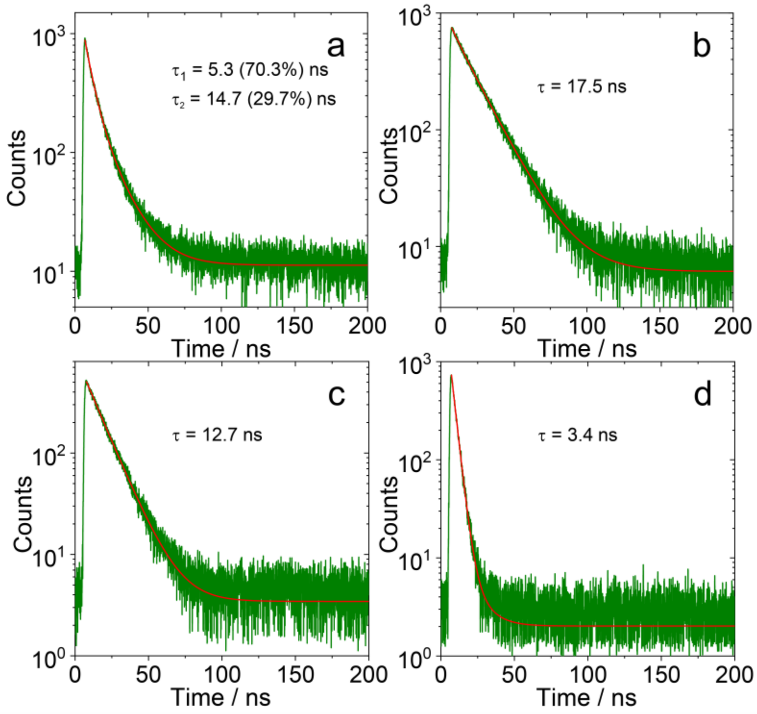

Fig. S12 Fluorescence decay traces of the **DPTZ-O-DTO** were monitored under different atmosphere (N_2, Air): (a) HEX at 455 nm, (b) DCM at 490 nm, (c) ACN at 550 nm and (d) MeOH at 560 nm, $c = 1.0 \times 10^{-5}$ M, λ_{ex} = 340 nm, 25 °C.

5. Femtosecond Transient Absorption (fs-TA) Spectra.

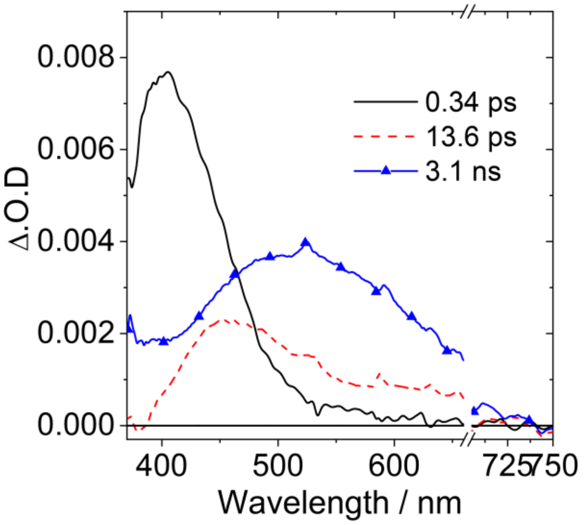

Fig. S13 EADS obtained from global analysis of the transient absorption data measured for **Br-DTO** in TOL with excitation at 350 nm.

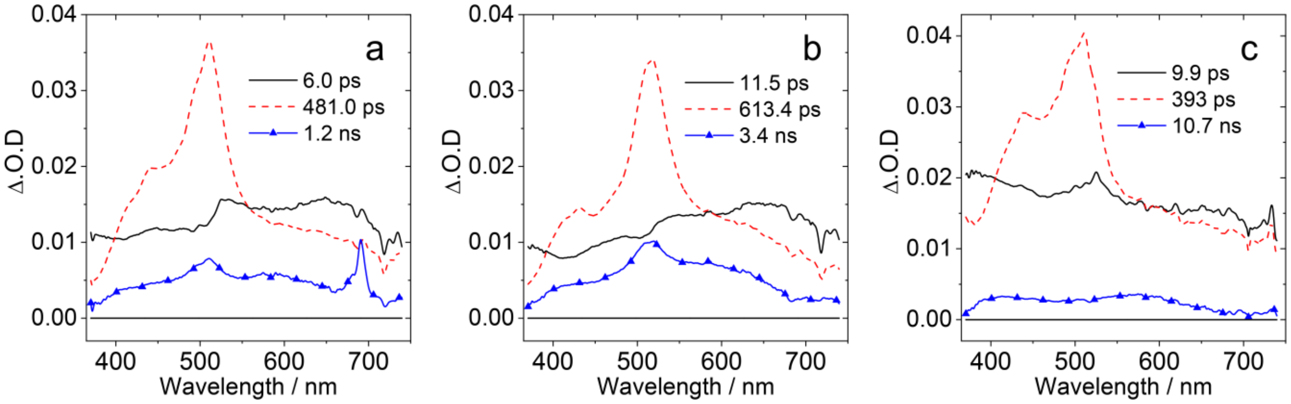

Fig. S14 EADS obtained from global analysis of the transient absorption data measured for (a) **PTZ-DTO**, (b) **PSeZ-DTO** and (c) **DPTZ-DTO** in benzonitrile (BZN) with excitation at 350 nm.

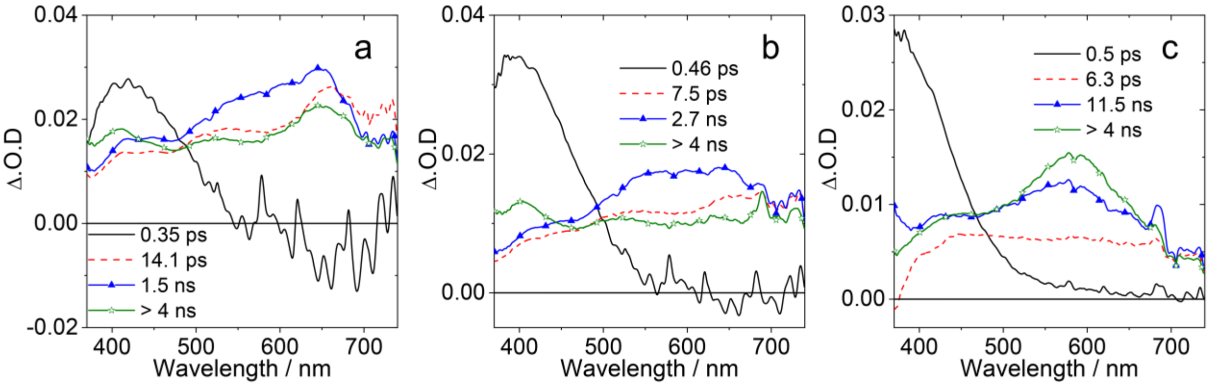

Fig. S15 EADS obtained from global analysis of the transient absorption data measured for (a) **PTZ-O-DTO**, (b) **PTZ-O2-DTO** and (c) **DPTZ-O-DTO** in BZN with excitation at 350 nm.

6. Spectroelectrochemistry.

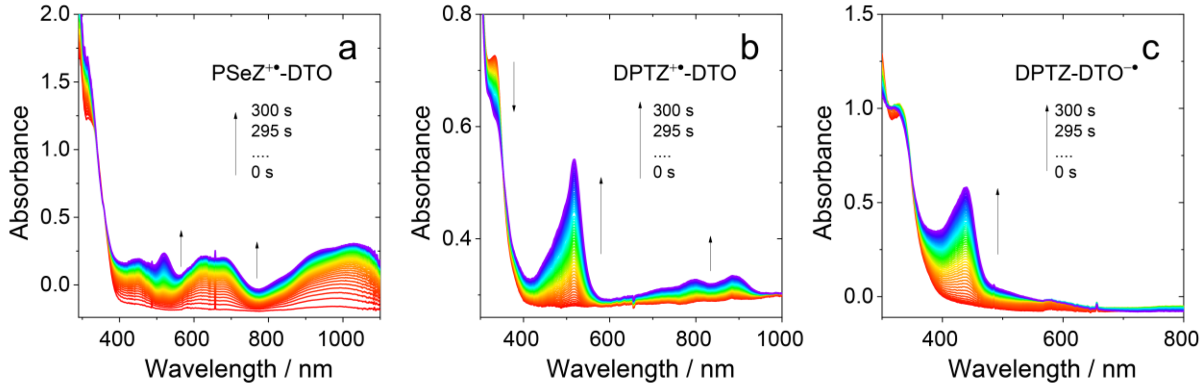

Fig. S16 The UV–vis absorption changes of (a) **PSeZ-DTO** upon oxidation under 0.48 V. **DPTZ-DTO** upon (b) oxidation under 0.58 V, (c) reduction under –2.09 V. The potentials are versus Ag/$AgNO_3$. The spectra were recorded in situ with a spectroelectrochemical cuvette (1 mm optical path) in deaerated DCM. 25 °C.

7. Nanosecond Transient Absorption (ns-TA) Spectra.

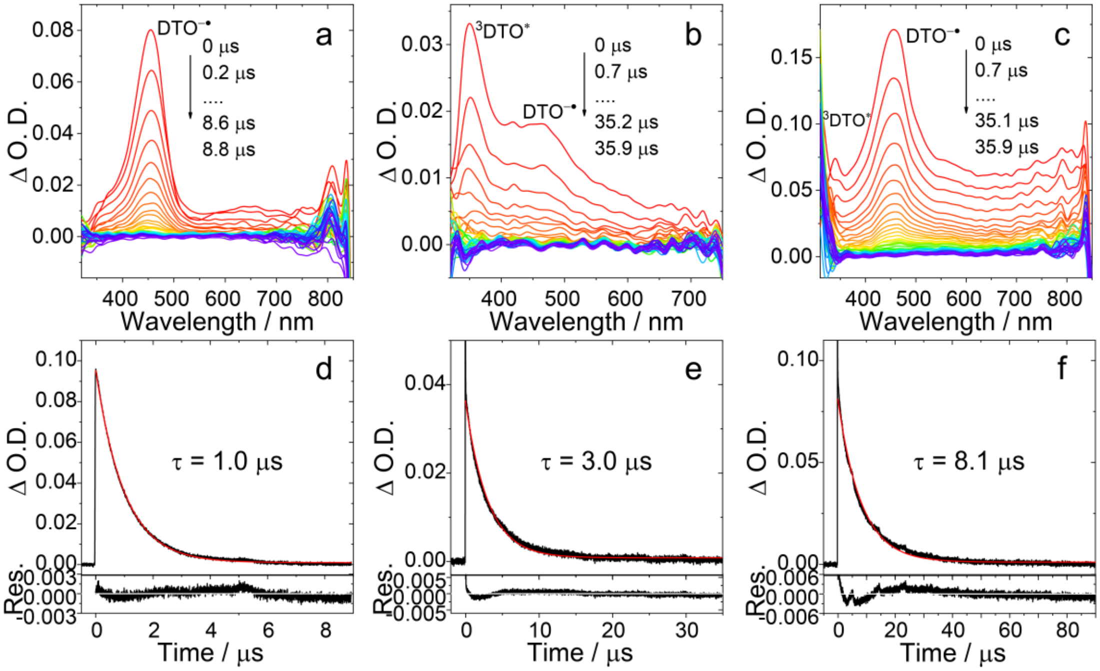

Fig. S17 Nanosecond transient absorption spectra of the dyads. Transient absorption spectra of (a) **PTZ-DTO**, (b) **PSeZ-DTO**, (c) **DPTZ-DTO**, and decay curves of (d) **PTZ-DTO**, (e) **PSeZ-DTO**, (f) **DPTZ-DTO** at 480 nm in deaerated HEX after pulsed laser excitation at 355 nm, $c = 2.0 \times 10^{-5}$ M, 25 °C.

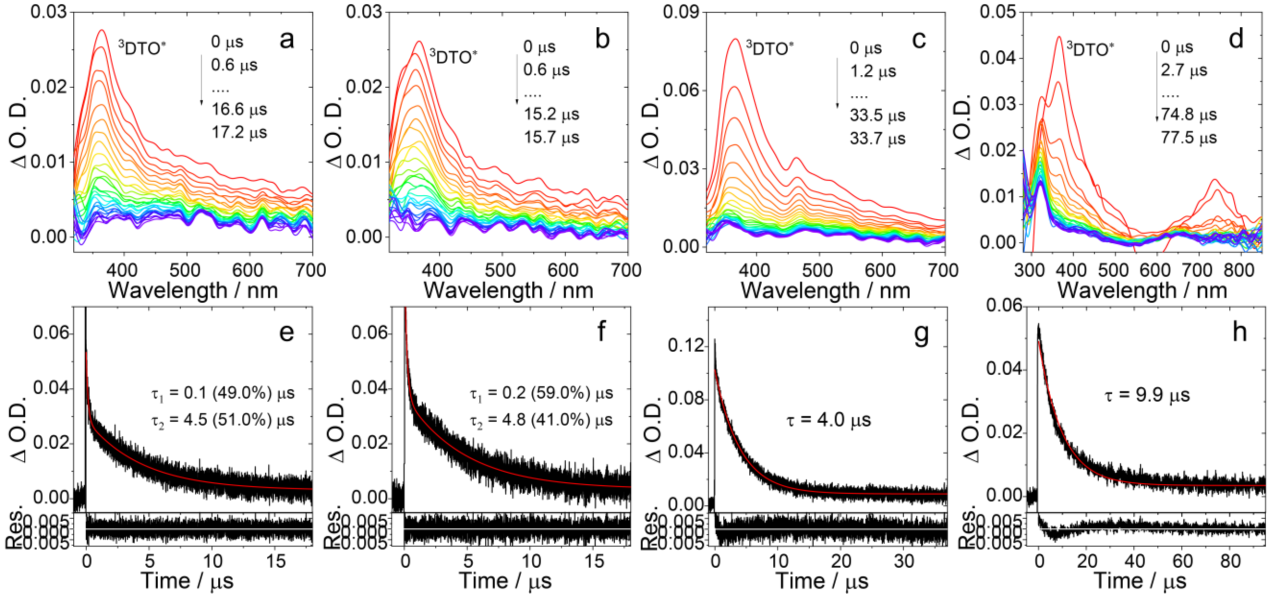

Fig. S18 Nanosecond transient absorption spectra of (a) **PTZ-O-DTO**, (b) **PTZ-O2-DTO**, (c) **DPTZ-O-DTO** and (d) **Br-DTO**. Decay curves of (e) **PTZ-O-DTO**, (f) **PTZ-O2-DTO**, (g) **DPTZ-O-DTO** at 370 nm in deaerated THF and (h) **Br-DTO** at 370 nm in deaerated DCM after pulsed laser excitation at 355 nm, $c = 2.0 \times 10^{-5}$ M, 25 °C.

8. Time-Resolved Electron Paramagnetic Resonance (TREPR) Spectra.

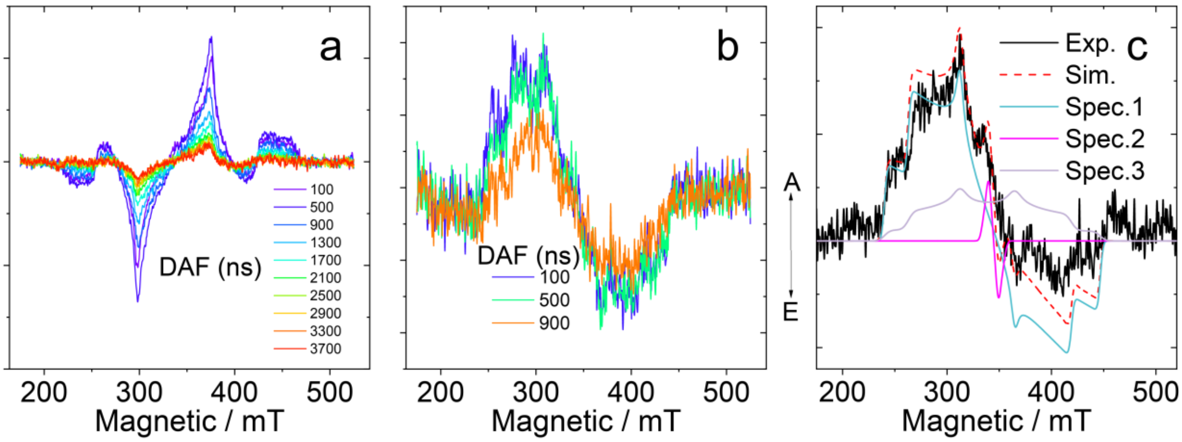

Fig. S19 TREPR spectra of **PTZ-DTO**. The laser excitation wavelength is 355 nm with energy 1 mJ; solvent is (a) TOL/2MeTHF (3/1); (b) DCM/2MeTHF (1/3). (c) Experimental and simulation in DCM/2MeTHF (1/3). Simulation spectrum is a sum of spectra of Spec1, Spec2 and Spec3, c = 5.0 × 10^{-4} M, 80 K.

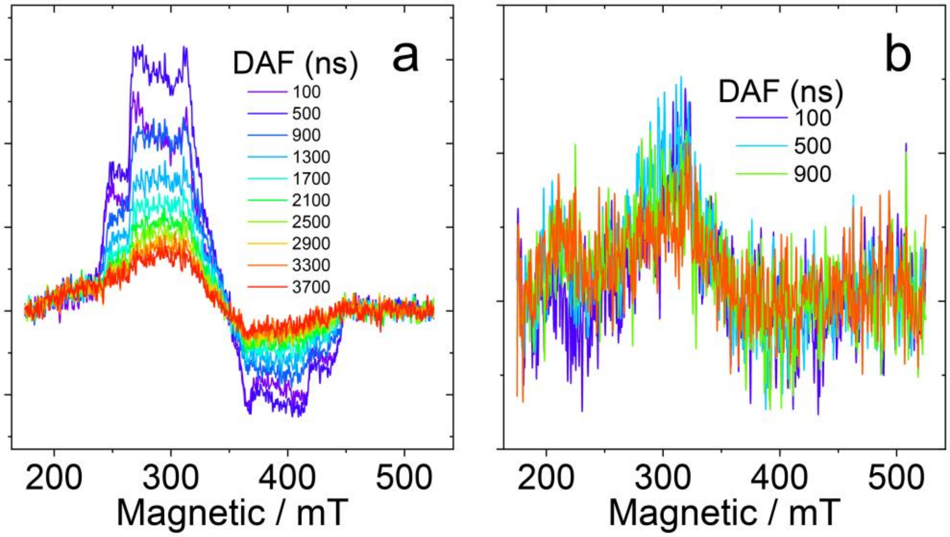

Fig. S20 TREPR spectra of **PSeZ-DTO**. The laser excitation wavelength is 355 nm with energy 1 mJ; solvent is (a) TOL/2MeTHF (3/1); (b) DCM/2MeTHF (1/3), c = 5.0 × 10^{-4} M, 80 K.

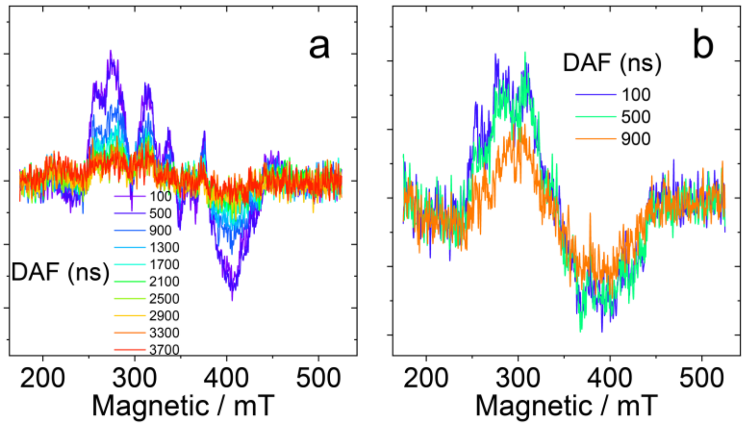

Fig. S21 TREPR spectra of **DPTZ-DTO**. The laser excitation wavelength is 355 nm with energy 1 mJ; solvent is (a) TOL/2MeTHF (3/1); (b) DCM/2MeTHF (1/3), $c = 5.0 \times 10^{-4}$ M, 80 K.

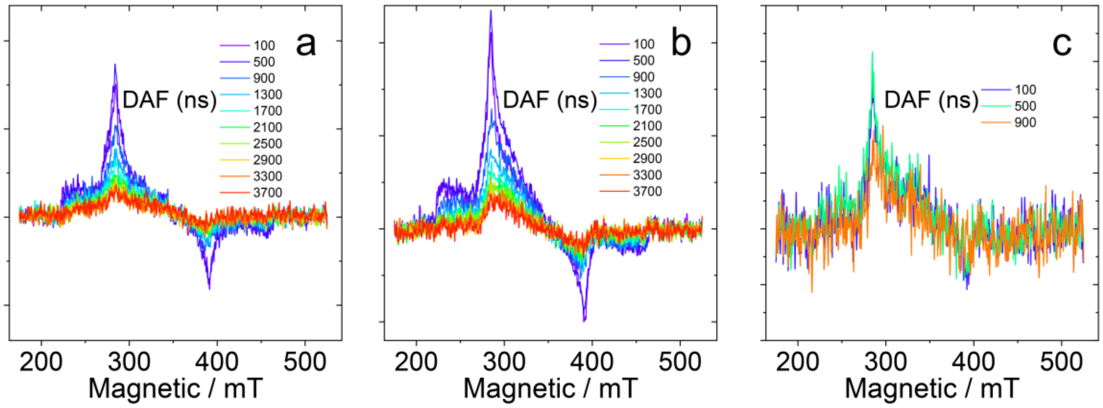

Fig. S22 TREPR spectra of (a) **PTZ-O-DTO**, (b) **PTZ-O2-DTO** and (c) **DPTZ-O-DTO**. The laser excitation wavelength is 355 nm with energy 1 mJ; solvent is TOL/2MeTHF (3/1), $c = 5.0 \times 10^{-4}$ M, 80 K.

Table S1. Zero field splitting parameters (*D* and *E*) and relative population rates p_x, p_y, p_z of the zero field spin states obtained from simulations of the triplet TREPR spectra of the PTZ-DTO[a]

Compound	$\|D\|$ (MHz); $\|E\|$ (MHz)	p_x: p_y: p_z
**PTZ-DTO**	[2900; 480], Spec 1	0.00: 0.00: 1.00
	–RP with dip-dip = –200 MHz ($D_Z^{dip\text{-}dip}$), Spec 2	T_0 triplet precursor
	[2900; 480], Spec 3	net-polarization

[a] Obtained from simulations of the triplet state TREPR spectra of the indicated molecule in DCM/2MeTHF (1/3) at 80 K.

9. DFT Calculations.

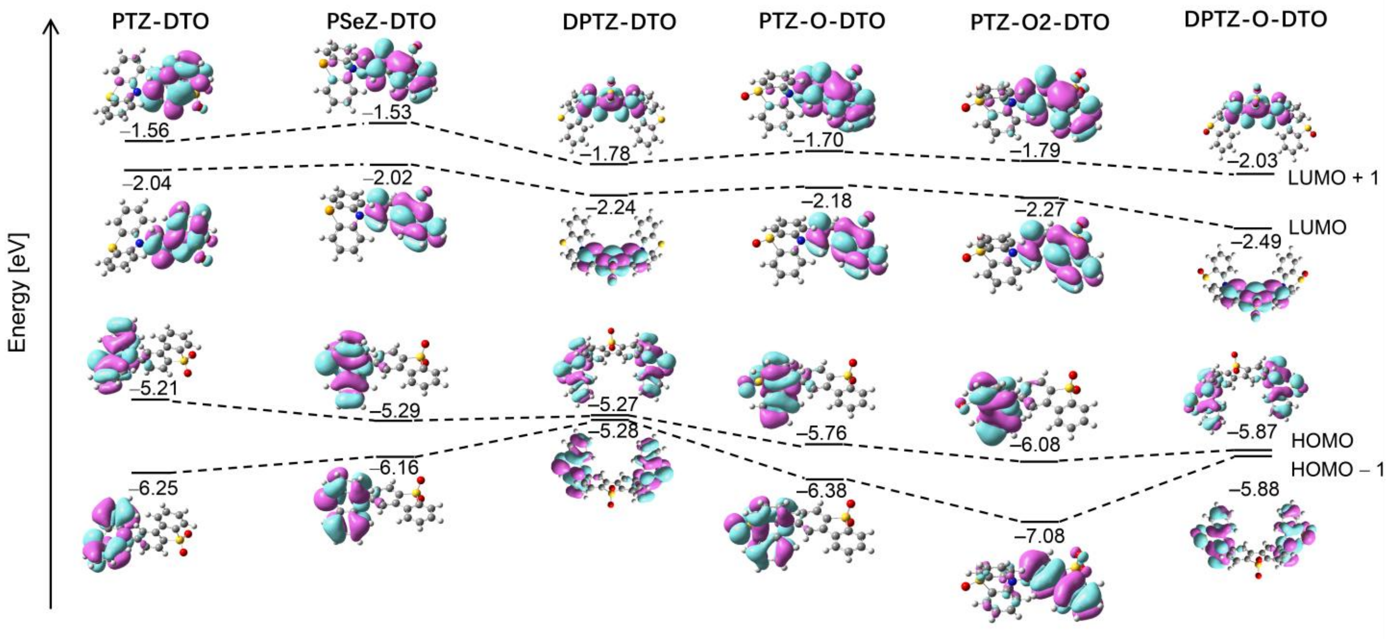

Fig. S23 Selected frontier molecular orbitals and the energy levels (eV) of the dyads calculated by DFT at the B3LYP/6-31G(d)(GENECP) level with Gaussian 16, based on the optimized ground state.

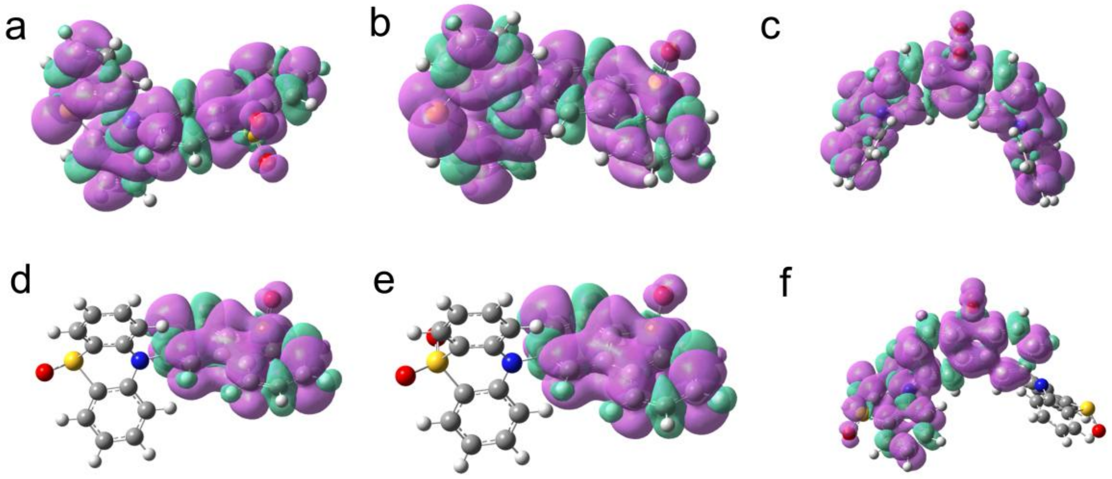

Fig. S24 Spin density surfaces of the dyads in the T_1 state of (a) **PTZ-DTO**, (b) **PSeZ-DTO**, (c) **DPTZ-DTO**, (d) **PTZ-O-DTO**, (e) **PTZ-O2-DTO** and (f) **DPTZ-O-DTO** in ACN, isovalue = 0.02. Calculations were performed by DFT at the B3LYP/6-31G(d) level with Gaussian 16.

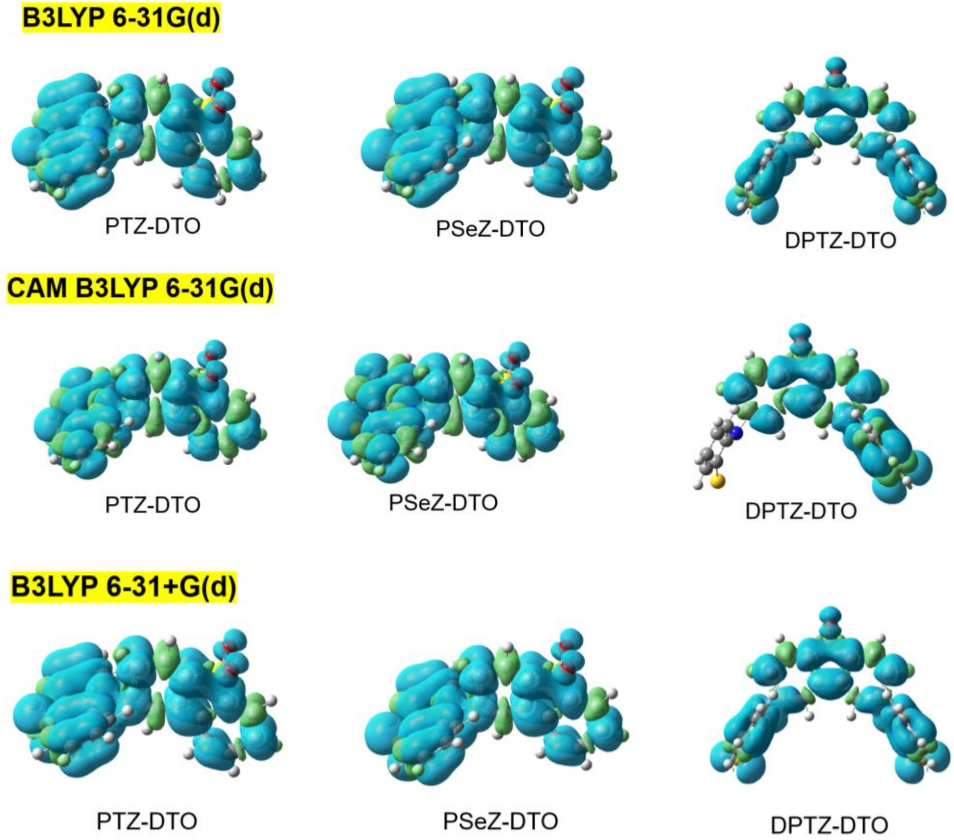

Fig. S25 Comparison of the Spin density surfaces of the dyads in the T_1 state of **PTZ-DTO**, **PSeZ-DTO** and **DPTZ-DTO** in TOL by using different functionals and basis sets (as indicated in the figure), isovalue = 0.02. Calculations were performed by DFT at the B3LYP/6-31G(d) level with Gaussian 16.

Table S2. Calculated ZFS *D* and *E* parameters of dyads.

Compound	Theory	*D* (cm^{-1}) [a]	*E*/*D*	Ref.
Cyclopentadienylidene Carbene	B3LYP/EPR-II	+0.426	0.016	This work
	B3LYP/IGLO-II	+0.442	0.024	This work
	B3LYP/ZORA-TZVP	+0.436	0.022	This work
	B3LYP/Def2-TZVP	+0.430	0.022	This work
	Experiment	+0.408	0.046	–[b]
**PTZ-DTO**	B3LYP/ZORA-TZVP	–0.462 (0.1134)	0.101	
**PSeZ-DTO**	B3LYP/ZORA-TZVP	–16.153 (0.0967)	0.159	
**DPTZ-DTO**	B3LYP/ZORA-TZVP	–0.321 (0.1134)	0.046	
**PTZ-O-DTO**	B3LYP/ZORA-TZVP	–0.197 (0.1134)	0.120	
**PTZ-O2-DTO**	B3LYP/ZORA-TZVP	–0.109 (0.1134)	0.124	
**DPTZ-O-DTO**	B3LYP/ZORA-TZVP	–0.108 (0.1134)	0.205	

[a] The numbers in the parenthesis are the experimental values presented in the main text of the manuscript (absolute values).
[b] Wolfgang Kirmse, Carbene Chemistry, 2nd Ed., Academic Press, July 10, 2013, ISBN: 9780323161459

Following the suggestion of the reviewer, calculations on *D* and *E* parameters were performed with ORCA 6.1.0.[10] Due to the limited time and resources, the calculations were performed at B3LYP/ZORA-TZVP level of theory with geometry of the T_1 state of dyads optimized at TD-B3LYP/Def2-TZVP level of theory. ZFS *D* and *E* were calculated with coupled-perturbed method with both spin-spin and spin-orbit parts included based on the spin density of spin-unrestricted natural orbital of excited dyads. Before start, benchmark calculations were performed on ZFS parameters of cyclopentadienylidene carbene. As the results obtained at level of B3LYP/ZORA-TZVP with ZORA relativistic hamiltonians is already comparable with those obtained at B3LYP/EPR-II and B3LYP/IGLO-II levels of theory as recommended by ORCA 5.0[10] and experiments, B3LYP/ZORA-TZVP is selected for these dyads. The calculated ZFS parameters are at

the same level as those obtained experimentally Tables 5 and S1. The larger |*D*| values found for **PTZ-DTO**, **DPTZ-DTO** and **DPTZ-DTO** can be attributed to the overestimation of spin-orbit coupling (SOC) of these dyads in the calculation.

We also calculated the orientation of the ZFS principle axis (Fig. S26). Since correlation of the experimental results of the population rates of the sublevels of the T_1 state, with the calculation result is not straight forward, because it requires the detail study of the ISC channels of the compounds, we did not move further in this aspect.

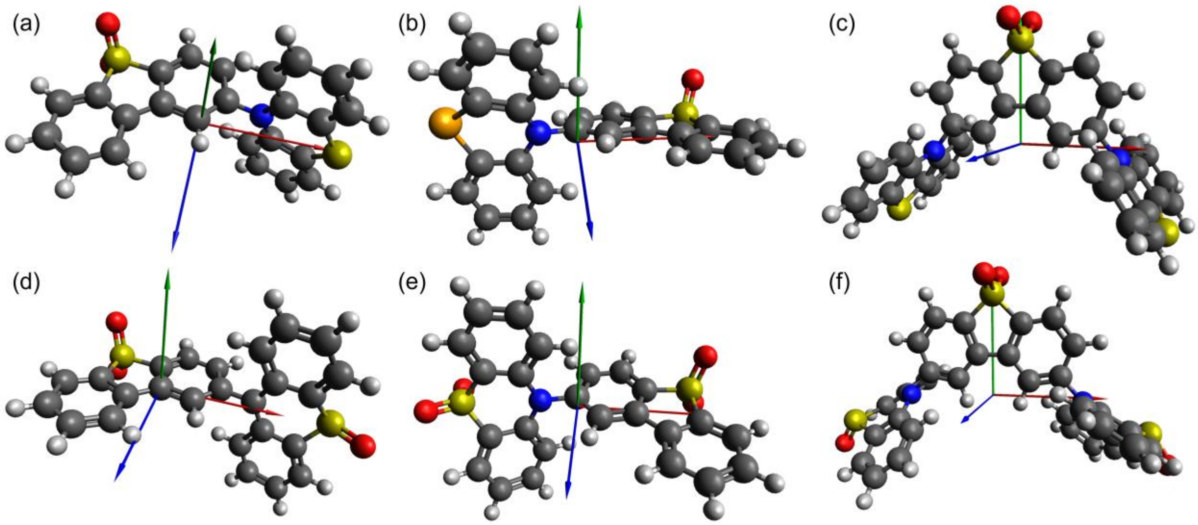

Fig. S26 Orientation of the ZFS tensor principal axes of PTZ-DTO(a), PSeZ-DTO(b), DPTZ-DTO(c), PTZ-O-DTO(d), PTZ-O2-DTO(e) and DPTZ-O-DTO(f). In (a)-(f), the C, H, S, Se, N and O are in dark gray, light gray, yellow, orange, blue and red, respectively, and the x, y and z axes are in red, green and blue, respectively.

10. References

1. Y. Rout, C. Montanari, E. Pasciucco, R. Misra and B. Carlotti, *J. Am. Chem. Soc.*, 2021, **143**, 9933–9943.

2. J. J. Snellenburg, S. Laptenok, R. Seger, K. M. Mullen, I. van Stokkum, *J. Stat. Software* **2012**, *49*, 1–22.

3. S. Stoll and A. Schweiger, *J. Magn. Reson*, 2006, **178**, 42–55.

4. M. J. Frisch, G. W. Trucks, H. B. Schlegel, G. E. Scuseria, M. A. Robb, J. R. Cheeseman, i. G. Scalmani, V. Barone, G. A. Petersson, H. Nakatsuji, et al., *Gaussian 16, Rev. C. 01*, Gaussian, Inc., Wallingford, CT. 2016.

5. A. D. Becke, "Density-functional thermochemistry. III. The role of exact exchange," *J. Chem. Phys.*, **1993**, *98*, 5648–52.

6. G. A. Petersson, A. Bennett, T. G. Tensfeldt, M. A. Al-Laham, W. A. Shirley, and J. Mantzaris, "A complete basis set model chemistry. I. The total energies of closed-shell atoms and hydrides of the first-row atoms," *J. Chem. Phys.*,

1988, *89*, 2193–218.

7. T. H. Dunning Jr. and P. J. Hay, in *Modern Theoretical Chemistry*, Ed. H. F. Schaefer III, Vol. 3 (Plenum, New York, 1977) 1–28.
8. A. Klamt, Conductor-like Screening Model for Real Solvents: A New Approach to the Quantitative Calculation of Solvation Phenomena. *J. Phys. Chem.* **1995**, *99*, 2224−2235.
9. T. N. Singh-Rachford and F. N. Castellano, *Coord. Chem. Rev.*, 2010, **254**, 2560–2573.
10. F. Neese, *Software Update: the ORCA Program System – Version 5.0*. *Wiley Interdiscip. Rev.: Comput. Mol. Sci.*, **2022**, *12*, 1, e1606
